# Antimalarial Activity of Acetylenic Thiophenes from *Echinops hoehnelii* Schweinf

**DOI:** 10.3390/molecules22111965

**Published:** 2017-11-21

**Authors:** Helen Bitew, Wendimagegn Mammo, Ariaya Hymete, Mariamawit Yonathan Yeshak

**Affiliations:** 1 Department of Pharmaceutical Chemistry and Pharmacognosy, School of pharmacy, College of Health Sciences, Addis Ababa University, Addis Ababa P.O. Box 9086, Ethiopia; helen.bitew@mu.edu.et (H.B.); hymete@yahoo.com (A.H.); 2 DDepartment of Pharmacognosy, School of Pharmacy, College of Health Sciences, Mekelle University, P.O.Box 1871, Ethiopia; 3 Department of Chemistry, College of Natural and Computational Sciences, Addis Ababa University, Addis Ababa P.O. Box 1176, Ethiopia; wendimagegn.mammo@aau.edu.et

**Keywords:** *Echinops hoehnelii* Schweinf, acetylenicthiophenes, in vivo antimalarial, acute oral toxicity

## Abstract

Malaria is one of the world’s most severe endemic diseases and due to the emergence of resistance to the currently available medicines, the need for new targets and relevant antimalarial drugs remains acute. The crude extract, four solvent fractions and two isolated compounds from the roots of *Echinops hoehnelii* were tested for their antimalarial activity using the standard four-day suppressive method in *Plasmodium berghei-*infected mice. The 80% methanol extract exhibited suppression of 4.6%, 27.8%, 68.5% and 78.7% at dose of 50, 100, 200 and 400 mg/kg respectively. The dichloromethane fraction displayed chemosuppression of 24.9, 33.5 and 43.0% dose of 100, 200 and 400 mg/kg of body weight. Five acetylenicthiophenes were isolated from the dichloromethane fraction of which 5-(penta-1,3-diynyl)-2-(3,4-dihydroxybut-1-ynyl)-thiophene decreased the level of parasitaemia by 43.2% and 50.2% while 5-(penta-1,3-diynyl)-2-(3-chloro-4-acetoxy-but-1-yn)-thiophene suppressed by 18.8% and 32.7% at 50 and 100 mg/kg, respectively. The study confirmed the traditional claim of the plant to treat malaria and could be used as a new lead for the development of antimalarial drugs.

## 1. Introduction

Malaria is still the world’s major important parasitic disease that is responsible for the death of more people than any other communicable disease except tuberculosis [1]. At least half a million deaths occur every year due to malaria, and about 90% of the cases of malaria worldwide occurred in Africa [2]. It is also a major public health problem in Ethiopia since approximately 75% of Ethiopia’s population lives in malarious areas and 2,118,815 confirmed malarial cases were reported in 2014 by WHO [3].

Treating the vast number of patients infected with malaria has resulted in widespread resistance to current antimalarial drugs [4]. Plants have been used as a source of medicine throughout history and continue to serve as the basis for many pharmaceuticals used today [5]. For that reason, there is a strong desire by many countries to be more active in using their ethnopharmacological heritage to identify new medicines to combat malaria [6].

*Echinops* L., which belongs to the family Asteraceae, is a large genus comprising about 120–130 species distributed across north and tropical Africa, the Mediterranean Basin, and central Asia. Majority of the plants are perennial with a few annuals [7,8]. Twelve of those species are found in Ethiopia of which *Echinops longisetus*, *E*. *ellenbeckii*, *E*. *kebericho* and *E*. *buhaitensis* are endemic [7].

Thiophenes have been isolated from the different species of *Echinops*. Investigation of the distribution of thiophenes in *Echinops* spp from Ethiopia resulted in isolation of 5-(but-3-en-1-ynyl)-2,2′′-bithiophene, α-terthiophene and other monothiophene derivatives [9]. Moreover, seven thiophenes; 5-(penta-1,3-diynyl)-2-(but-3-en-1-ynyl)-thiophene, 5-(penta-1,3-diynyl)-2-(4-acetoxy-but-1-ynyl)-thiophene, 5-(penta-1,3-diynyl)-2-(3-hydroxy-4-acetoxy-but-1-ynyl)-thiophene, 5-(penta-1,3-diynyl)-2-(3,4-diacetoxy-but-1-ynyl)-thiophene, 5-(penta-1,3-diynyl)-2-(3-chloro-4-acetoxy-but-1-ynyl)-thiophene, 5-(penta-1,3-diynyl)-2-(3,4-epoxy-but-1-ynyl)-thiophene and 5-[(5-acetoxymethyl -2-thienyl)-2-(but-3-en-1-ynyl)]-thiophene have been reported from roots of *E*. *ellenbeckii* [10].

*E*. *hoehnelii* is a perennial shrub which can grow up to 1.5 m in height with dark brown stem, sharp triangular ultimate segments, basal or lower cauline leaves up to 45 × 22 cm and heads 3–4 cm in diameter. It grows on stream banks with altitude of 2050–3750 m. It is found in Eastern African countries including Ethiopia, Democratic Republic of Congo, Rwanda and Burundi [7]. Thiophenes are the only secondary metabolites reported from this plant [9].

The root of this plant is traditionally used to treat malaria in Sheko ethnic group in southern Ethiopia [11]. Nevertheless, there is no scientific evidence to support this claim. Hence, in the present study the root extract of *E*. *hoehnelii* has been investigated for antimalarial activity.

## 2. Results and Discussion

### 2.1. Acute Oral Toxicity

No gross behavioral changes, weight loss and mortality were observed in the experimental animals within 24 h and for the next fourteen days after administration of single fixed dose (2 g/kg) of the 80% methanol root extract. Hence, it could be deduced that the LD_50_ of the crude root extract of *E*. *hoehnelii* was greater than 2 g/kg.

### 2.2. Antimalarial Activity of 80% Methanol Root Extract of E. hoehnelii

The result of this study signified that 80% methanol root extract of *E*. *hoehnelii* possessed dose-dependent antimalarial activity with significant chemosuppression in *Plasmodium berghei*-infected mice (Table 1). Except for the lowest dose, i.e., 50 mg/kg, the extract showed significant suppression (*p* < 0.01) compared to the negative control. The standard drug, chloroquine (CQ), which was used as a positive control resulted in complete eradication of the parasite. The survival time of the mice treated with the extract also improved; the 400 mg/kg dose resulted in statistically significant difference (*p* < 0.05) compared to the negative control. The relatively low survival time of the mice treated with the crude extract of *E. hoehnelii* might be due to reoccurrence of the disease which results in early death of animals [12].

As shown in Table 2, the 80% methanol extract prevented weight loss of infected mice at all doses. The weight of the mice didn’t exhibit statistically significant difference before and after treatment of the extract at all doses. The difference in percentage change wasn’t also statistically significant among the groups treated with the extract and compared to the negative control.

### 2.3. Antimalarial Activity of Fractions

The four solvent fractions were tested for antimalarial activity to identify the active principles responsible for the activity of the crude extract, through bioassay guided fractionation. According to the results, the dichloromethane fraction showed statistically significant suppression of parasitemia at all doses compared to the negative control (*p* < 0.001). The highest activity was attained at 400 mg/kg (Table 3). The dichloromethane fraction also prevented weight loss (Table 4) and improved survival time. The 400 mg/kg dose displayed statistically significant difference (*p* < 0.05) compared to the negative control.

The hexane fraction of the crude extract displayed chemosuppression that increased with dose (Table 3). The survival time of mice treated with this fraction was also improved, of which 200 mg/kg and 400 mg/kg showed statistically significant difference compared to the negative control. In spite of low chemosuppression activity, the survival time of mice treated with the hexane fraction was higher than of the negative control. Besides, all the doses of hexane fraction prevented weight loss. This suggested that the fractions of the plant extract may possess other benefits to the host such as acting as analgesics, antipyretics or as immune stimulators other than direct parasiticidal effects [13].

The ethyl acetate fraction displayed a chemosuppression in non-dose-dependent manner. The middle dose (200 mg/kg) showed better suppression than of the lower and upper doses used in this study. The survival time of the mice treated with this fraction was not statistically different from the negative control. Similar to the ethyl acetate fraction, the parasitemia of mice treated with aqueous fraction wasn’t significantly different from the negative control. The aqueous fraction improved the survival time compared to the negative control even though significant difference between the treated and the untreated groups was not observed. The weight of mice treated with aqueous fraction increased on the fifth day of infection in accordance with the activity. Generally, the activity displayed by the ethyl acetate and aqueous fractions wasn’t significant. The activity of the aqueous fraction decreased with dose. Similar findings were also reported for the aqueous root extract of *Lecaniodis cuscupanioides* [14].

### 2.4. Isolation and Structural Elucidation of Compounds from the Dichloromethane Fraction

**Figure 1:**
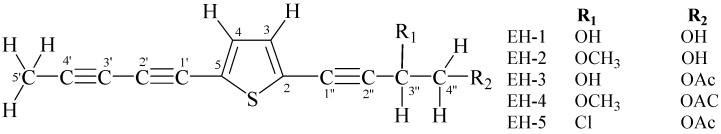
Acetylenicthiophenes from *Echinops hoehnelii* Schweinf.

The dichloromethane fraction was taken for further analysis in the effort to isolate and identify active principles of *E*. *hoehnelii*. The HPLC profile of the fraction revealed the presence of five major compounds. This fraction was subjected to open column chromatography over silica gel. The purification of the sub fractions by PTLC yielded five compounds coded as EH-1, EH-2, EH-3, EH-4 and EH-5 (Figure 1). The structural elucidation of the compounds was conducted by using the spectroscopic and spectrometric data and by comparing with exisiting literature (Supplementary Material). EH-1 and EH-2, have been previously reported from *Pulchea indica* [15] where as Abegaz et al. isolated EH-3 and EH-5 from the roots of *E*. *hoehnelii* [9]. EH-4, which is a closely related compound to EH-2, EH-3 and EH-7, has not been mentioned elsewhere to the best of the knowledge of the authors.

### 2.5. Antimalarial Activity of Isolated Compounds

EH-1 and EH-5, the two major compounds of the dichloromethane fraction, were screened for antimalarial activity. Two different doses of EH-1 were administered and both doses decreased the level of parasitaemia significantly (*p* < 0.001) compared to the negative control (Table 5). The survival time of the *P. berghei-*infected mice treated with EH-1 was also significantly different (*p* < 0.05) from that of the negative control. The weight of the mice treated with EH-1 increased; but the measures taken before and after treatment weren’t significantly different (Table 6). EH-5 which contains an ester functional group displayed lower suppression at 50 mg/kg and 100 mg/kg of body weight compared to EH-1. This compound showed significant activity (*p* < 0.01) only at 100 mg/kg while the survival time of mice treated with both doses didn’t show a significant difference compared to the negative control (Table 5).

The major compounds have been tested for antimalarial activity. EH-1 has been previously reported to possess antimicrobial activity against *Saccharomyces cerevisiae*, *Staphylococcus aureus* and *Bacillus subtilis* [15]. The induction property of EH-1 on NQO1, an enzyme that is important for detoxification pathway, has also been described [16]. The structural isomer of EH-1 isolated from *Pluchea indica*, has been reported to decease the level of parasitaemia from 7% to 1.01% and 1.8% in *P*. *berghei* and *P*. *yoelii* infected mice administered with 10 mg/kg [17]. Antifungal activity against *Colletotrichum gloeosporioides* and *Fusarium oxysporum* of EH-5 has also been reported [18].

## 3. Materials and Methods

### 3.1. General

The UV spectra were recorded on Perkin-Elmer Lambda 950 UV/VIS/SNR (Perkin Elmer, Boston, MA, USA) spectrometer using methanol as a solvent. The positive ion high-resolution ESI mass spectra were obtained from Orbitrap Elite mass spectrometer (Thermofisher Scientific, Darmstadt, Germany) equipped with a HESI (Heated Electro spray Ionization) electro spray ion source (positive spray voltage 3.5 kV; capillary temperature 275 °C, source heater temperature 200 °C; FTMS resolution 30.000). IR spectra were recorded on Perkin-Elmer 65 IR spectrometer (Perkin Elmer, Boston, MA, USA). NMR spectra were recorded at room temperature on BrukerAvance 400 FT-NMR spectrometer (Avance DMX 400, München, Germany) operating at 400.13 for ^1^H and 100.6 MHz for ^13^C using deuterated chloroform.

The dichloromethane fraction and isolated compounds were analyzed by Analytical HPLC (Agilent HPLC 1260 infinity with G-1311B quaternary, Waldbronn, Germany) coupled with UV/visible detector (G1314F VWD) at 280 nm. A reversed phase C18, 4.6 × 100 mm column (Agilent Eclipse Plus, Santa Clara, CA, USA) with a particle size of 3.5 µm was used. The solvent system involved gradient elution starting from 70% methanol in water to 100% methanol with flow rate of 0.3 mL/min by increasing the proportion of methanol by 5% within 5 min. Samples were dissolved in methanol and injected into the HPLC system at 25 °C.

### 3.2. Plant Material

The roots of *E*. *hoehnelii* were collected on 9 March 2014 from Bale Mountains National Park, Ethiopia. Authenticity of the plant material was confirmed by taxonomists of the National Herbarium, the Department of Plant Biology and Biodiversity Management, Addis Ababa University, where voucher specimen (collection number HB 001) was deposited.

### 3.3. Experimental Animals and Parasite

Swiss albino mice of either sex weighing 20–29 g and 6–8 weeks of age were obtained from Ethiopian Public Health Institute (EPHI) animal house. The animals were kept at room temperature with 12 h light/dark cycle providing free access to standard pellets and water. They were acclimatized for a week before the study and, the experiments were conducted in accordance to the internationally accepted laboratory animal use, care and guideline [18]. Ethical clearance was obtained from the School Ethics Review Board: clearance number ERB/SOP/41/10/2016. *Plasmodium berghei* (Chloroquine sensitive strain) obtained from EPHI was used. The parasites were maintained by serial passage of blood from infected to non-infected mice on weekly basis.

### 3.4. Preparation of Extract and Fractions

The roots of the plant were properly cleaned, air dried in the shed at room temperature, and crushed in to coarse powder. The powdered plant material (1.2 kg) was macerated with 80% methanol for 72 h with occasional shaking. The mixture was then filtered using filter paper and the mark was re-macerated (2×). The filtrate was combined and concentrated under reduced pressure in a rotary evaporator (Buchi Rota Vapor R-200, Flawil, Switzerland)a temperature not exceeding 40 °C, and then dried in freeze dryer (Operon, Gimpo-si, Korea). The dried extract was transferred into vials and kept in a desiccator until future use.

The 80% methanol extract (20 g) was suspended in of distilled water (200 mL) and partitioned twice using equal volumes n-hexane, dichloromethane and ethyl acetate. The fractions were concentrated under reduced pressure using rotary evaporator (Buchi Rota Vapor R-200, Flawil, Switzerland) and dried in oven (Gallenkamp, London, UK) at a temperature less than 40 °C. The dried fractions were stored in a desiccator until further use.

### 3.5. Isolation of Compounds

The dichloromethane fraction was subjected to open column chromatography over silica gel (particle size of 0.2–0.5 mm) using chloroform and chloroform: ethyl acetate mixtures of increasing polarity. The progress of separation was monitored by analytical thin layer chromatography (TLC) over pre-coated silica gel plates.

Preparative TLC was carried out on silica gel coated glass plates (20 cm × 20 cm) with thickness of 0.25 mm. The solvent systems used were either chloroform: ethyl acetate (95:5) or chloroform: ethyl acetate (1:1). The developed chromatograms were visualized under UV light (254 nm). Bands of the compounds were scrapped off, washed with methanol: ethyl acetate (1:1) solution and filtered to yield five compounds.

EH-1: yellow amorphous solid (*t_R_* = 12.10 min); UV (MeOH) λ_max_ 247, 319, 340 nm; IR (neat) ν_max_ 1079, 2150, 2924, 3335 cm^−1^; ^1^H-NMR (CDCl_3_, 400.13 MHz) δ 7.064 (1H, d, *J* = 4 Hz, H-3), 7.125 (1H, d, *J* = 4 Hz, H-4), 2.06 (3H, s, H-5′), 4.705 (1H, dd, *J*_1_ = 4 Hz, *J*_2_ = 6.4, H-3′′), 3.794 (1H, dd, *J*_1_ = 4 Hz, *J*_3_ = 11.2 Hz, H-4′′a), 3.854 (1H, dd, *J*_2_ = 6.4 Hz, *J*_3_ = 11.2 Hz, H-4′′b); ^13^C-NMR (CDCl_3_, 100.6 MHz) δ 124.19 (C, C-2),132.41 (C, C-3), 133.55 (C, C-4), 123.75 (C, C-5), 66.35 (C, C-1′), 79.59 (C, C-2′), 64.10 (C, C-3′), 83.58 (C, C-4′), 4.80 (C, C-5′), 78.93 (C, C-1′′), 91.38 (C, C-2′′), 63.78 (C, C-3′′), 66.22 (C, C-4′′); EIMS *m*/*z* 253.0298 [M + Na]^+^ (calcd. for C_13_H_10_O_2_S, 253.029922).

EH-2: yellow amorphous solid (*t_R_* = 14.558 min); UV (MeOH) λ_max_ 247, 317, 340 nm; IR (neat) ν_max_1110, 2235, 2922, 3437 cm^−1^; ^1^H-NMR (CDCl_3_, 400.13 MHz) δ 7.072 (1H, d, *J* = 4 Hz, H-3), 7.130 (1H, d, *J* = 4 Hz, H-4), 2.06 (3H, s, H-5′), 4.305 (1H, t, *J* = 5.6 Hz, H-3′′), 3.812 (2H, d, *J* = 6 Hz, H-4′′), 3.55 (3H, s, OCH_3_); ^13^C-NMR(CDCl_3_, 100.6 MHz) δ 124.09 (C, C-2), 132.4 (C, C-3), 133.54 (C, C-4), 123.88 (C, C-5), 66.38 (C, C-1′), 79.53 (C, C-2′), 64.11 (C, C-3′), 83.54 (C, C-4′), 4.80 (C, C-5′), 79.89 (C, C-1′′), 89.60 (C, C-2′′), 72.68 (C, C-3′′), 65.00 (C, C-4′′), 57.12 (C, OCH_3_); EIMS *m*/*z* 267.0456 [M + Na]^+^ (calcd. for C_14_H_12_O_2_S, 267.045572).

EH-3: brown amorphous solid (*t_R_* = 15.35 min); UV (MeOH) λ_max_ 247, 318, 340 nm; IR (CCl_4_) ν_max_ 785,1040, 1227, 1750, 2231, 2926, 3444 cm^−1^; ^1^H-NMR (CDCl_3_, 400.13 MHz) δ 7.13 (1H, d, *J* = 4 Hz, H-3), 7.06 (1H, d, *J* = 4 Hz, H-4), 2.06 (3H, s, H-5′), 4.84 (1H, dd, *J* = 6.4, 4.4 Hz, H-3′′), 4.30 (1H, dd, *J* = 11.4, 6.4 Hz, H-4′′a), 4.34 (1H, dd, *J* = 11.4, 4.4 Hz, H-4′′b); ^13^C-NMR (CDCl_3_, 100.6 MHz) δ 124.34 (C, C-2), 133.54 (C, C-3), 132.49 (C, C-4), 123.60 (C, C-5), 64.09 (C, C-1′), 66.31 (C, C-2′), 79.64 (C, C-3′), 67.17 (C, C-4′), 4.80 (C, C-5′), 79.09 (C, C-1′′), 90.53 (C, C-2′′), 61.73 (C, C-3′′), 67.17 (C, C-4′′), 170.93 (C, C=O), 20.80 (C, -COCH_3_) EIMS *m*/*z* 295.0404 [M + Na]^+^ (calcd. for C_15_H_12_O_3_S, 295.040487).

EH-4: brown amorphous solid (*t_R_* = 18.85); UV (MeOH) λ_max_ 247, 319, 340 nm; IR(neat) _max_, 786, 1045, 1230, 1753, 2228, 2918 cm^−1^; ^1^H-NMR (CDCl_3_, 400.13 MHz) δ 7.075 (1H, d, *J* = 3.6 Hz, H-3), 7.133 (1H, d, *J* = 3.6 Hz, H-4), 2.065 (3H, s, H-5′), 4.459 (1H, dd, *J*_1_ = 4 Hz, *J*_2_ = 7 Hz, H-3′′), 4.293 (1H, dd, *J*_1_ = 4 Hz, *J*_3_ = 11.5 Hz, H-4′′a), 4.342 (1H, dd, *J*_2_ = 7 Hz, *J*_3_ = 11.5 Hz, H-4′′b); ^13^C-NMR (CDCl_3_, 100.6 MHz) δ 124.22 (C, C-2),132.4557 (C, C-3), 133.53 (C, C-4), 123.74 (C, C-5), 66.35 (C, C-1′), 79.58 (C, C-2′), 64.11 (C, C-3′), 83.38 (C, C-4′), 4.83 (C, C-5′), 80.0 (C, C-1′′), 88.91 (C, C-2′′), 69.96 (C, C-3′′), 65.33 (C, C-4′′); EIMS *m*/*z* 309.0559 [M + Na]^+^ (calcd. for C_16_H_14_O_3_S, 309.056137).

EH-5: brown amorphous solid (*t_R_* = 21.22 min); UV (MeOH) λ_max_ 247, 321, 341 nm; IR (CCl_4_) ν_max_ 1045, 1230, 1753, 2228, 2918 cm^−1^; ^1^H-NMR(CDCl_3_, 400.13 MHz) δ 7.137 (1H, d, *J* = 4 Hz, H-3), 7.106 (1H, d, *J* = 4 Hz, H-4), 2.066 (3H, s, H-5′), 4.966 (1H, dd, *J*_1_ = 5.6 Hz, *J*_2_ = 7.2 Hz, H-3′′), 4.398 (1H, dd, *J*_2_ =7.2 Hz, *J*_3_ = 9.6 Hz, H-4′′a), 4.463 (1H, dd, *J*_1_ = 5.6 Hz, *J*_3_ = 9.6 Hz, H-4′′b); ^13^C-NMR (CDCl_3_, 100.6 MHz) δ 124.89 (C, C-2), 133.05 (C, C-3), 133.56 (C, C-4), 123.05 (C, C-5), 66.20 (C, C-1′), 79.89 (C, C-2′), 64.07 (C, C-3′), 83.79 (C, C-4′), 4.84 (C, C-5′), 80.22 (C, C-1′′), 88.16 (C, C-2′′), 46.08 (C, C-3′′), 66.43 (C, C-4′′), 170.34 (C, C=O), 20.71 (C, -COCH_3_).

### 3.6. Acute Oral Toxicity Test

Acute oral toxicity test was conducted on the 80% methanol root extract of *E. hoehnelii* according to the procedure of OECD guideline 425 [19,20]. Five non-pregnant and nulliparous female albino mice were used for the test. All the animals were fasted for 3 h before and 1 h after the administration of the extract. Accordingly, one animal was administered 2 g/kg of the crude extract dissolved in 2% Tween 80 in distilled water and additional four animals were administered 24 h later. The animals were observed continuously for 4 h immediately after administration and once daily for 14 consecutive days. Animals were observed for gross changes such as loss of appetite, hair erection, lacrimation, tremors, convulsions, salivation, diarrhea, mortality and other signs of over toxicity [19,20].

### 3.7. Antimalarial Activity Test of 80% Methanol Root Extract of E. hoehnelii

The standard four-day suppressive method developed by Peter et al. (1975) [21] was used to determine the in vivo antimalarial activity of the 80% methanol extract. Blood was taken from a donor mouse with approximately 30% parasitemia and diluted with physiological saline to 5 × 10^7^ parasitized erythrocytes per mL. Thirty mice were infected with 0.2 mL of blood at day zero intraperitoneally (i.p.) and randomly divided in to six groups of 5 animals. Groups I-IV were treated with 50, 100, 200 and 400 mg/kg of the crude extract in 2% Tween 80, while group V and VI were treated with chloroquine (25 mg/kg) and the vehicle, respectively. All test substances and vehicle were administered orally at a maximum volume of 0.5 mL using standard gavages. The treatment was started 3 h post-infection of the parasite and continued for 3 consecutive days once daily. At day 4, thin smears of blood film taken from the tail of each animal were prepared on two microscopic slides. The blood smears were fixed with methanol and stained with 10% Gemsa for 15 min. The slides were examined under the microscope (Olympus 6V20WHA2, Tokyo, Japan) with 100× magnifying power using oil immersion. Percentage parasitaemia and suppression was calculated according to the formula given below.

$$\%\;\mathrm{Parasitemia}=\frac{Number\;of\;parasitized\;RBC}{Total\;number\;of\;RBC\;count}\times 100$$

$$\%\;\mathrm{Suppression}=\frac{Parasitemia\;in\;negative\;control-Parasitemia\;in\;study\;group}{Parasitemia\;in\;negative\;control}\times 100$$

The weights of the mice were taken at first and on the fifth day to evaluate the weight loss protective effect of the extract [22,23].

### 3.8. Antimalarial Activity Test of the Fractions

Antimalarial activity of the four fractions was conducted according to the method mentioned in Section 2.5. The hexane, dichloromethane, ethyl acetate and aqueous fractions were dissolved in 2% Tween 80. Each fraction was orally administered at doses of 100, 200 and 400 mg/kg while vehicle and chloroquine (25 mg/kg) were given to the negative and positive control groups, respectively.

### 3.9. Antimalarial Activity of Isolated Compounds

Antimalarial activity of isolated compounds obtained from the dichloromethane fraction was conducted with the method described in Section 2.5. The major compounds, (5-(penta-1,3-diynyl)-2-(3,4-dihydroxybut-1-ynyl)-thiophene) (EH-1) and (5-(penta-1,3-diynyl)-2-(3-chloro-4-acetoxy-but-1-ynyl)-thiophene) (EH-5) were first dissolvedin 7% Tween 80/3% ethanol in distilled water. The mice in the test groups were treated with each of the test solutions at doses of 50 mg/kg and 100 mg/kg while the positive and the negative control groups were given chloroquine (25 mg/kg) and the vehicle, respectively.

### 3.10. Data Analyses

Results of the antimalarial study were expressed as mean ± SD (Standard deviation). The data were analyzed with Windows SPSS Version 16 using one-way analysis of variance (ANOVA) followed by Tukey’s HSD post-hoc test to compare the results between and among groups. Paired *t*-test was used to compare parameters before and after treatment. Differences were considered significant at *p* < 0.05.

## 4. Conclusions

The 80% methanol root extract of *E*. *hoehnelii* didn’t show acute oral toxicity which may indicate the safety of the plant. The crude extract as well as the fractions exhibited significant antimalarial activity in *P. berghei* mouse model. The results obtained support the traditional use of the plant. Phytochemical analysis resulted in isolation of acetylenicthiophenes. Two of the isolated compounds, i.e., EH-1 and EH-5 were found to show significant suppression of the parasite. The antimalarial activity of *E. hoehnelii* could then be attributed to the presence of the acetlylenic thiophenes; however further phytochemical and pharmacological analysis on trace compounds that could be possibly active needs to be done.

## Figures and Tables

**Table 1:** Percentage suppression and mean survival time of *Plasmodium berghei-*infected mice after administration of 80% methanol root extract of *Echinops hoehnelii*.

Dose	% Parasitaemia ± SD	% Suppression	Mean Survival Time (Days) ± SD
Vehicle	24.2 ± 2.3	-	6.4 ± 0.9
50 mg/kg	23.1 ± 2.5	4.6 ^b3d1e3f3^	7 ± 0.7 ^b3^
100 mg/kg	17.5 ± 5.4	27.8 ^a2b3c1e3f3^	7.4 ± 0.5 ^b3^
200 mg/kg	7.6 ± 0.9	68.5 ^a3b2c3d3^	7.8 ± 1.1 ^b3^
400 mg/kg	5.2 ± 1.2	78.7 ^a3c3d3^	8 ± 0.7 ^a1b3^
CQ (25 mg/kg)	0 ± 0.00	100 ^a3c3d3e2^	>28 ^a3c3d3e3f3^

Values are presented as M ± SD, *n* = 5; ^a^ = compared to vehicle; ^b^ = compared to CQ; ^c^ = compared to 50 mg/kg; ^d^ = compared to 100 mg/kg; ^e^ = compared to 200 mg/kg; ^f^ = compared to 400 mg/kg; ^1^ = *p* < 0.05; ^2^ = *p* < 0.01; ^3^ = *p* < 0.001.

**Table 2:** Body weight of *Plasmodium berghei-*infected mice before and after administration of 80% methanol root extract of *Echinops hoehnelii*.

Dose	Wt (g) D_0_ ± SD	Wt (g) D_4_ ± SD	% Change
Vehicle	25.6 ± 0.3	24.9 ± 1.1	−2.5
50 mg/kg	25.0 ± 0.2	25.7 ± 2.1	2.9
100 mg/kg	25.1 ± 0.3	25.7 ± 1.2	2.5
200 mg/kg	25.6 ± 0.2	26.1 ± 0.6	2.3
400 mg/kg	24.9 ± 0.7	25.6 ± 0.3	3.0
CQ (25 mg/kg)	23.9 ± 0.3	26.8 ± 0.6	12.0 ^3^

Values are presented as M ± SD; *n* = 5, Wt D_0_ = weight day zero; Wt D_4_ = weight day four; ^3^ = *p* < 0.001, comparison was made between weight before and after treatment.

**Table 3:** Percentage suppression and mean survival time of *Plasmodium berghei-*infected mice after administration of solvent fractions.

Fraction	Dose (mg/kg/Day)	% Parasitaemia ± SD	% Suppression	Mean Survival Time (Days) ± SD
Hexane	100	28.2 ± 11.7	11.6 ^b3^	7.2 ± 0.8 ^b3^
Hexane	200	27.8 ± 1.5	12.9 ^b3^	8.2 ± 0.8 ^a2b3^
Hexane	400	22.6 ± 3.3	29.1 ^b3^	8.2 ± 0.8 ^a2b3^
Vehicle	0.5 mL	31.9 ± 3.00	0	6.4 ± 0.5
CQ	25	0.00 ± 0.00	100 ^a3c3d3e3^	>28 ± 0.00 ^a3c3d3e3^
Dichloromethane	100	32.3 ± 2.5	24.8 ^a3b3e3^	8.0 ± 1.9 ^b3^
Dichloromethane	200	28.6 ± 1.8	33.5 ^a3b3e3^	8.4 ± 1.9 ^b3^
Dichloromethane	400	24.5 ± 1.5	43.0 ^a3b3c3d1^	9.0 ± 0.7 ^a1b3^
Vehicle	0.5 mL	43.00 ± 2.9	0	7.0 ± 1.6
CQ	25	0.00 ± 0.00	100 ^a3c3d3e3^	>28 ± 0.00 ^a3c3d3e3^
Ethyl acetate	100	37.3 ± 7.3	9.8 ^b3^	7.25 ± 1.3 ^b3^
Ethyl acetate	200	37.0 ± 1.6	10.5 ^b3^	7.0 ± 1.3 ^b3^
Ethyl acetate	400	39.4 ± 2.1	4.80 ^b3^	6.4 ± 0.9 ^b3^
Vehicle	0.5 mL	41.4 ± 4.0	0.00	6.54 ± 1.3
CQ	25	0.00 ± 0.00	100 ^a3c3d3e3^	>28 ± 0.00 ^a3c3d3e3^
Aqueous	100	33.2 ± 4.0	19.8 ^b3^	7.6 ± 1.3 ^b3^
Aqueous	200	38.1 ± 15.1	7.8 ^b3^	6.80 ± 1.3 ^b3^
Aqueous	400	40.0 ± 1.4	3.3 ^b3^	6.80 ± 0.1 ^b3^
Vehicle	0.5 mL	41.4 ± 4.0	0.00	6.54 ± 1.3
CQ	25	0.00 ± 0.00	100 ^a3c3d3e3^	>28 ± 0.00 ^a3c3d3e3^

Values are presented as M ± SD; *n* =5; ^a^ = compared to vehicle; ^b^ = compared to CQ; ^c^ = compared to 100 mg/kg; ^d^ = compared to 200 mg/kg; ^e^ = compared to 400 mg/kg; ^1^ = *p* < 0.05; ^2^ = *p* < 0.01; ^3^ = *p* < 0.001.

**Table 4:** Body weight of *Plasmodium berghei-*infected mice before and after administration of solvent fractions.

Fraction	Dose (mg/kg/Day)	Wt (g) D_0_ ± SD	Wt (g) D_4_ ± SD	% Change
Hexane	100	25.6 ± 0.2	26.0 ± 0.4	1.5
Hexane	200	25.5 ± 1.3	26.2 ± 0.5	2.4
Hexane	400	25.4 ± 0.3	26.3 ± 1.1	3.7
Vehicle	0.5 mL	26.2 ± 0.2	24.5 ± 0.5	−6.3 ^2^
CQ	25	25.4 ± 0.5	26.5 ± 0.3	4.2 ^2^
Dichloromethane	100	28.3 ± 3.0	29.0 ± 1.5	2.6
Dichloromethane	200	26.8 ± 3.4	27.7 ± 3.7	3.5
Dichloromethane	400	25.9 ± 3.3	27.1 ± 3.7	4.7
Vehicle	0.5 mL	27.9 ± 3.00	26.3 ± 4.4	−5.8
CQ	25	25.4 ± 0.5	26.5 ± 0.3	4.2 ^2^
Ethyl acetate	100	26.2 ± 0.7	26.4 ± 1.6	0.6
Ethyl acetate	200	25.0 ± 2.5	26.1 ± 2.5	4.3
Ethyl acetate	400	25.6 ± 2.6	26.5 ± 3.1	3.4
Vehicle	0.5 mL	26.8 ± 3.5	26.1 ± 5.9	−2.5
CQ	25	28.8 ± 5.1	29.8 ± 4.5	3.6
Aqueous	100	25.8 ± 2.1	26.7 ± 1.8	3.3
Aqueous	200	25.1 ± 2.5	25.7 ± 2.4	2.8
Aqueous	400	27.2 ± 2.3	27.9 ± 2.5	2.5
Vehicle	0.5 mL	26.8 ± 3.5	26.1 ± 5.9	−2.5
CQ	25	28.8 ± 5.1	29.8 ± 4.5	3.6

Values are presented as M ± SEM; *n* = 5, Wt D_0_ = weight day zero; Wt D_4_ = weight day four; ^2^ = *p* < 0.001, comparison was made between weight before and after treatment.

**Table 5:** Percentage suppression and mean survival time of *Plasmodium berghei-*infected mice after administration of EH-1 and EH-5.

Test	Dose	%Parasitaemia ± SD	% Suppression	Mean Survival Time (Days) ± SD
EH-1	50 mg/kg	23.5 ± 2.1	43.2 ^a3b3^	9.4 ± 1.5 ^a1b3^
EH-1	100 mg/kg	15.3 ± 8.7	50.2 ^a3b3^	9.7 ± 1.5 ^a1b3^
Vehicle	0.5 mL	30.8 ± 6.5	0	7.2 ± 1.2
CQ	25 mg/kg	0.00 ± 0.00	100 ^a3c3d3^	>28 ^a3c3d3^
EH-5	50 mg/kg	28.0 ± 3.2	18.8 ^b3^	7.2 ± 1.0 ^b3^
EH-5	100 mg/kg	23.2 ± 5.3	32.7 ^a2b3^	7.8 ± 1.3 ^b3^
Vehicle	0.5 mL	34.4 ± 3.9	0	7.2 ± 0.8
CQ	25 mg/kg	0.00 ± 0.00	100 ^a3c3d3^	>28 ^a3c3d3^

Values are presented as M ± SD; *n* = 5; ^a^ = compared to vehicle; ^b^ = compared to CQ; ^c^ = compared to 50 mg/kg; ^d^ = compared to 100 mg/kg; ^1^ = *p* < 0.05; ^2^ = *p* < 0.01; ^3^ = *p* < 0.001.

**Table 6:** Body weight of *Plasmodium berghei-*infected mice before and after administration of EH-1 and EH-5.

Test	Dose	Wt(g) D_0_ ± SD	Wt (g) D_4_ ± SD	% Change
EH-1	50 mg/kg	26.3 ± 2.4	26.6 ± 1.6	1.1
EH-1	100 mg/kg	22.4 ± 1.9	22.6 ± 1.6	0.8
Vehicle	0.5 mL	23.5 ± 1.6	23.1 ± 1.8	−1.5
CQ	25 mg/kg	27.6 ± 2.5	28.8 ± 2.7	4.4 ^1^
EH-5	50 mg/kg	22.0 ± 1.0	22.2 ± 2.1	0.9
EH-5	100 mg/kg	24.2 ± 2.6	25.4 ± 3.0	4.9
Vehicle	0.5 mL	24.0 ± 3.3	23.6 ± 2.9	−1.7
CQ	25 mg/kg	21.8 ± 1.6	24.1 ± 2.6	10.6 ^1^

Values are presented as M ± SD; *n* = 5, Wt D_0_ = weight day zero; Wt D_4_ = weight day four; ^1^ = *p* < 0.05, comparison was made between weight before and after treatment.

## Supplementary Materials

The List of spectrum are available online.
