# Supplementary material for: Antimalarial Activity of Acetylenic Thiophenes from Echinops hoehnelii Schweinf
Source: Molecules. 2017 Nov 21;22(11):1965. doi: 10.3390/molecules22111965 (PMC6150322; doi:10.3390/molecules22111965)
Supplement: Supplementary file 1 [file molecules-22-01965-s001.pdf]

List of spectrum

Appendix I: MS, IR, UV, ^{1}H NMR, ^{13}C NMR, DEPT-135 and HMBC spectra of EH-1

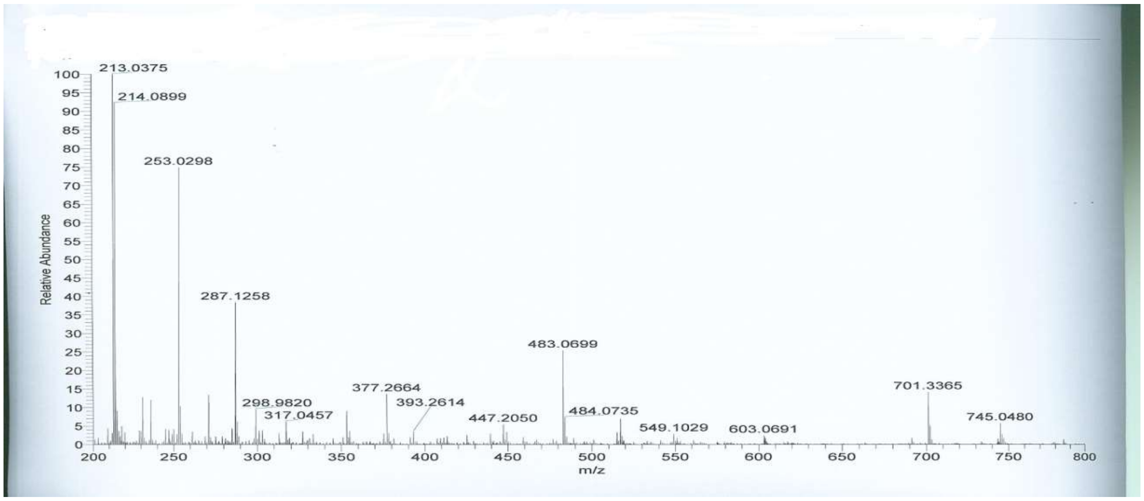

Figure A: MS spectrum of EH-1.

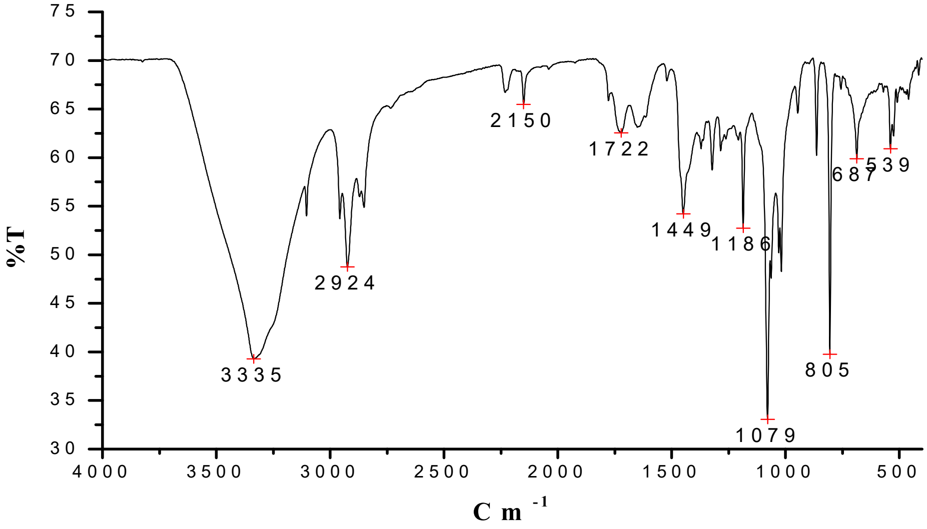

Figure B: IR spectrum of EH-1.

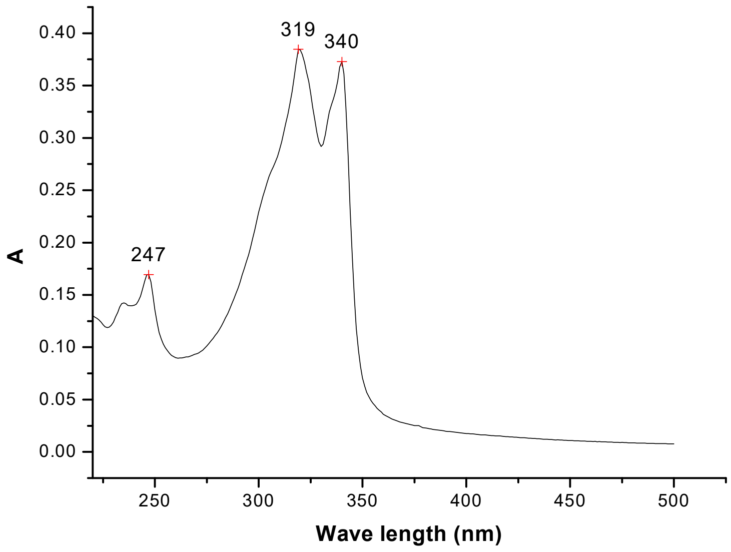

Figure C: UV spectrum of EH-1.

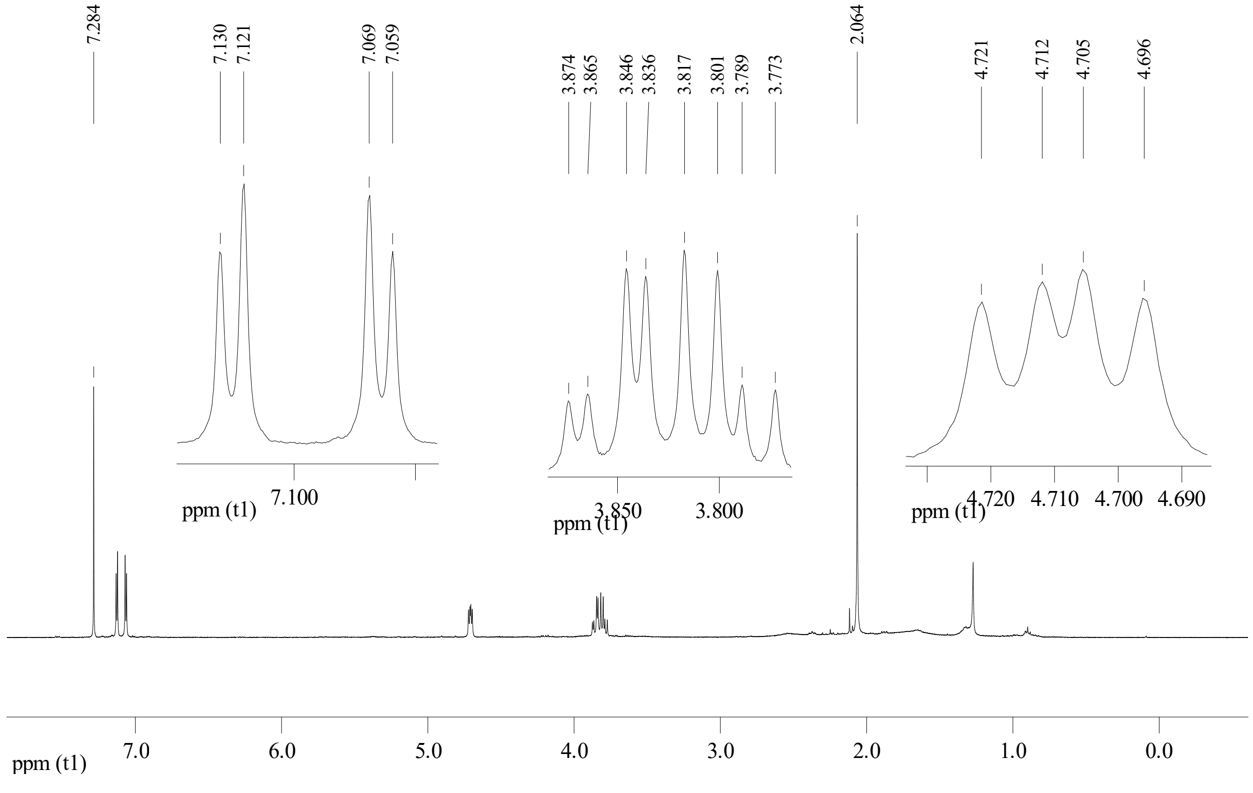

Figure D: ^{1}H NMR spectrum of EH-1.

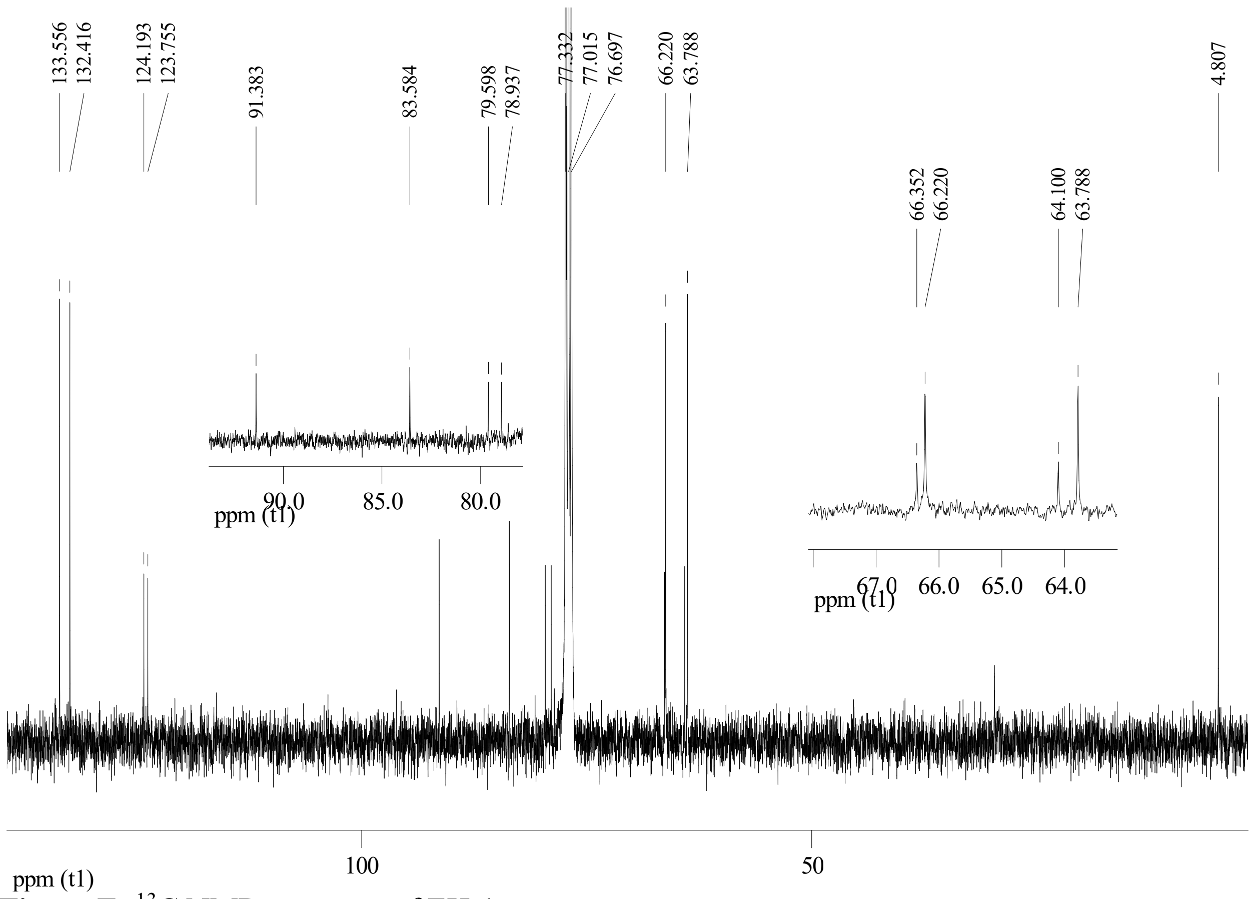

Figure E: ^{13}C NMR spectrum of EH-1.

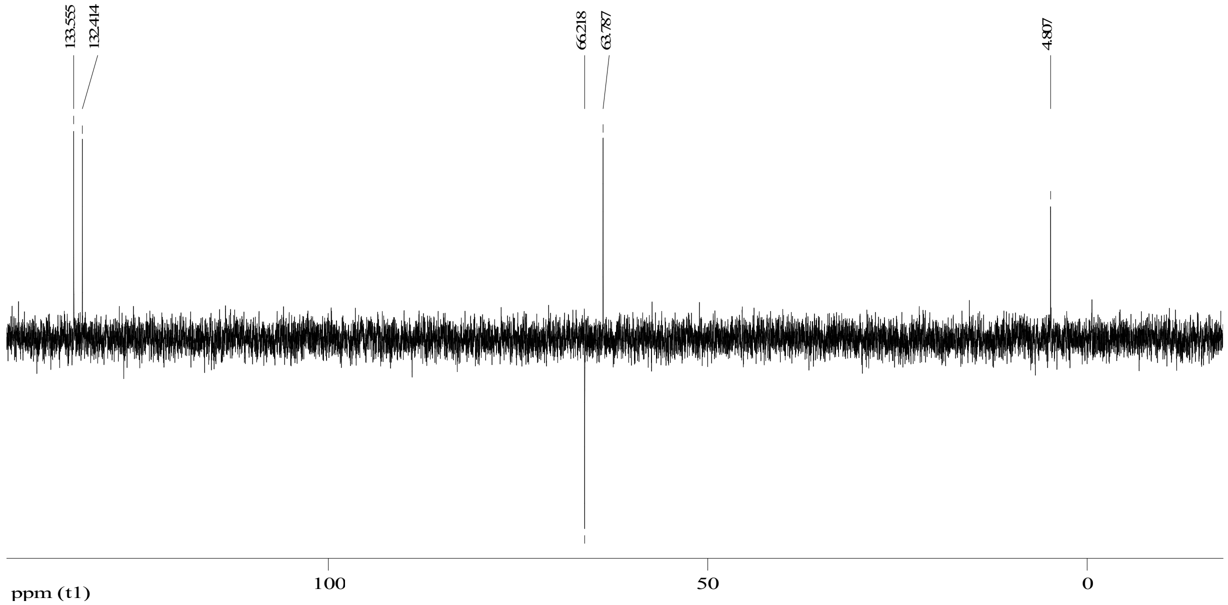

Figure F: DEPT-135 spectrum of EH-1.

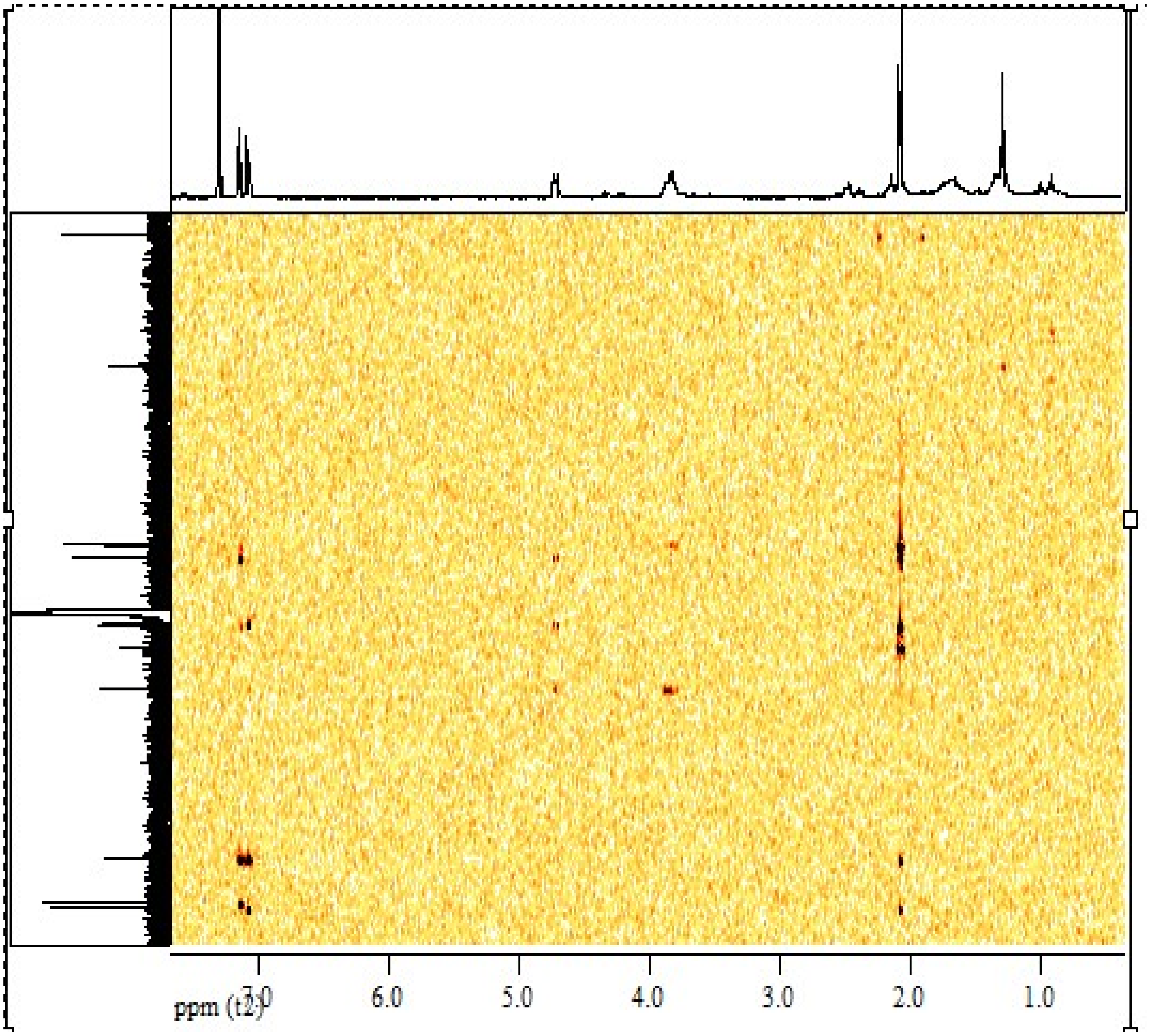

Figure G: HMBC spectrum of EH-1.

Appendix II: MS, IR, UV, ^{1}H NMR, ^{13}C NMR and DEPT-135 spectra of EH-2

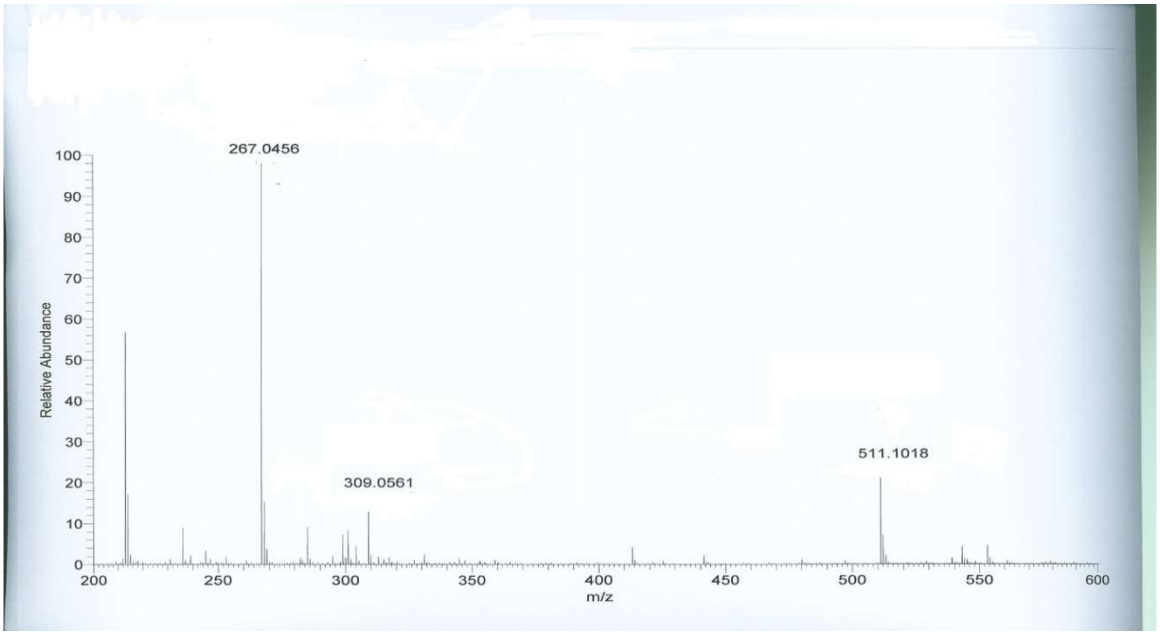

Figure H: MS spectrum of EH-2.

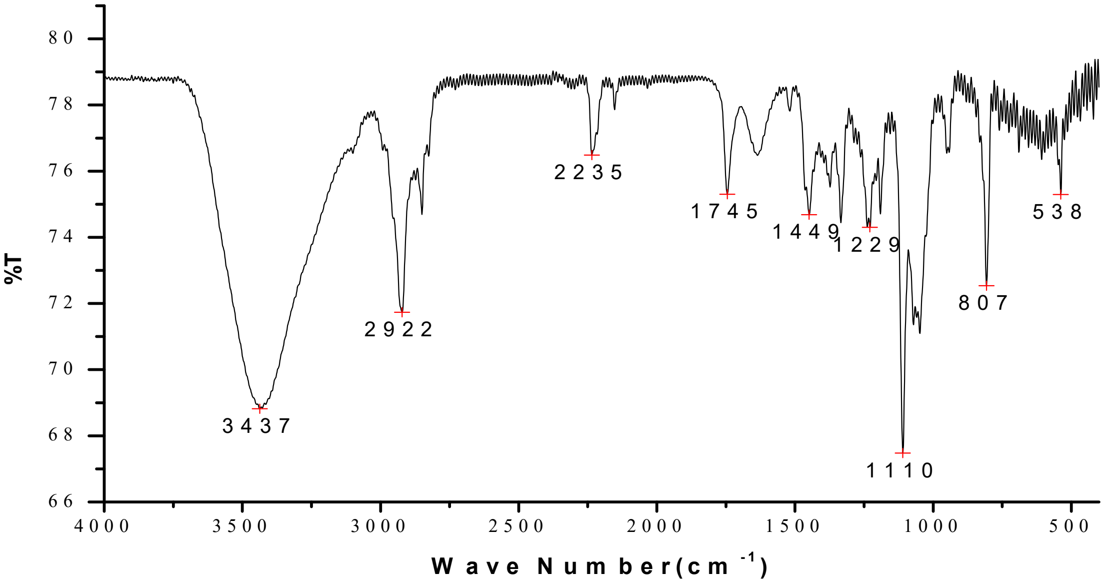

Figure I: IR spectrum of EH-2.

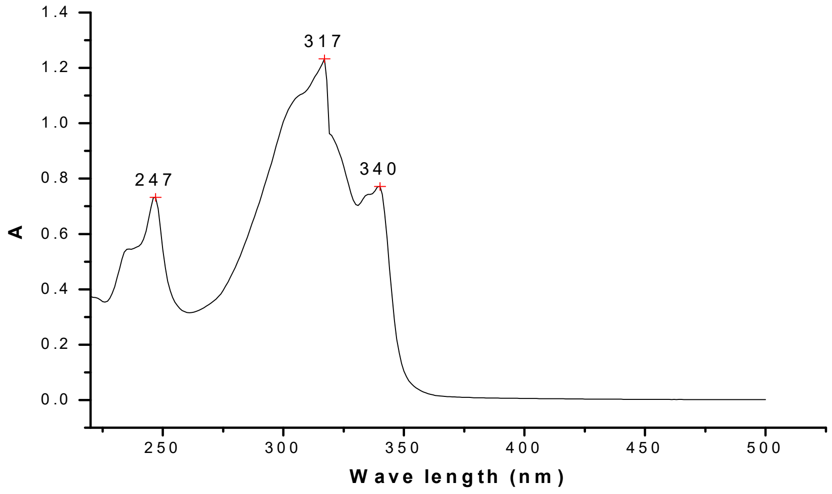

Figure J: UV spectrum of EH-2.

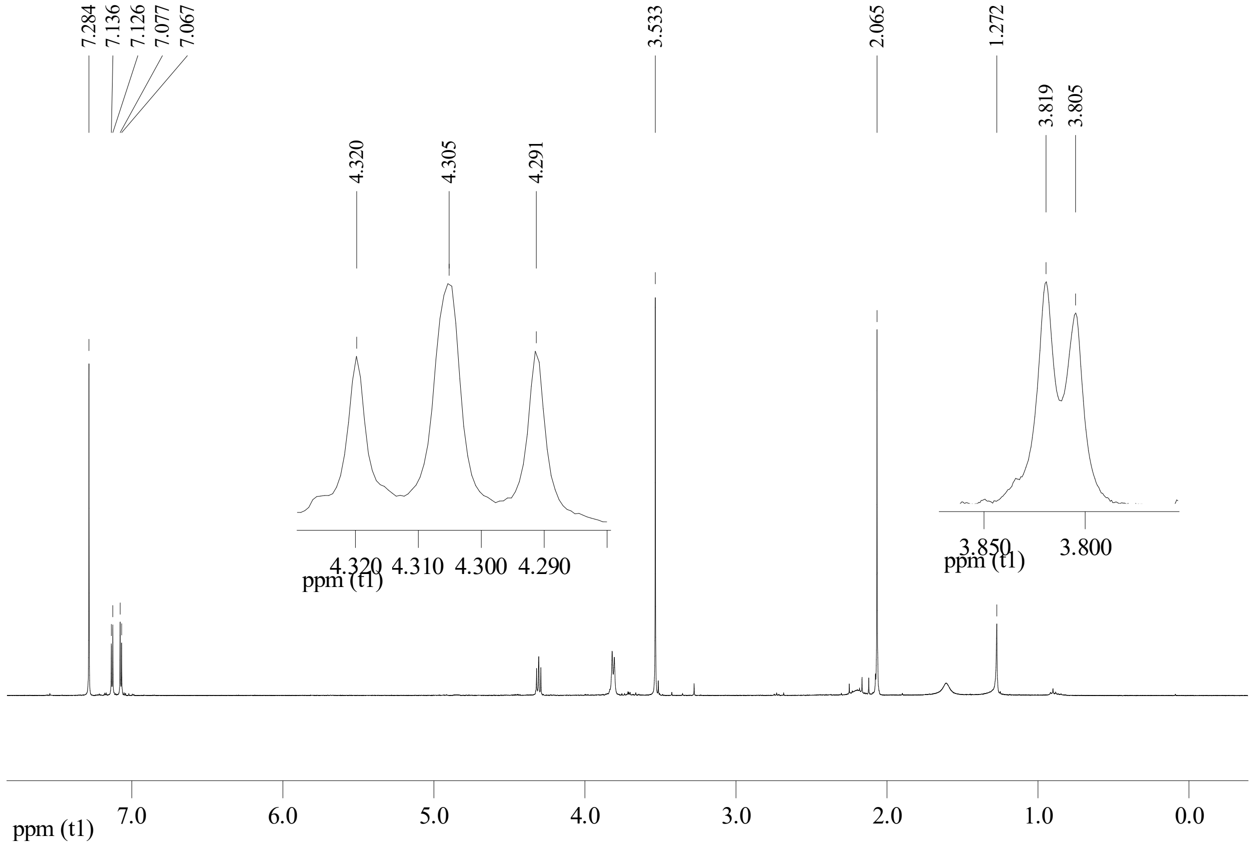

Figure K: ^{1}H NMR spectrum of EH-2.

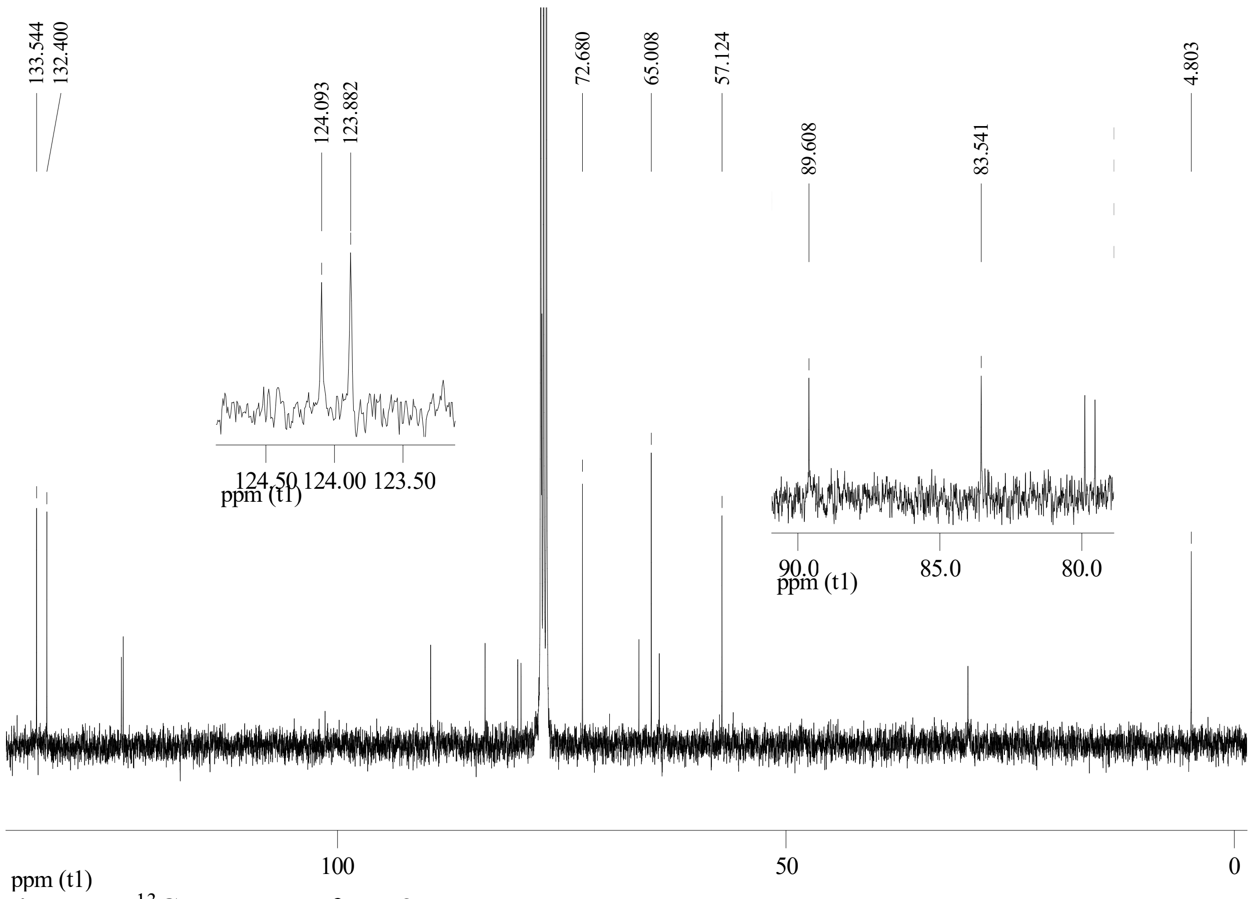

Figure L: ^{13}C spectrum of EH-2.

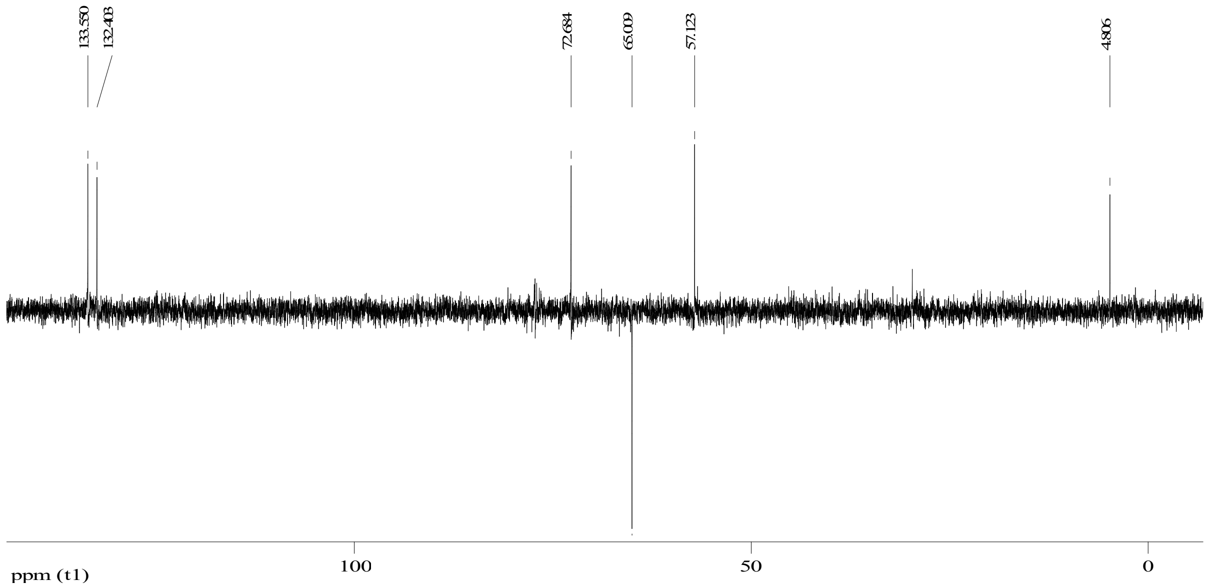

Figure M: DEPT-135 spectrum of EH-2.

Appendix IV: MS, IR, UV, ^{1}H NMR, ^{13}C NMR and DEPT-135 spectra of EH-3

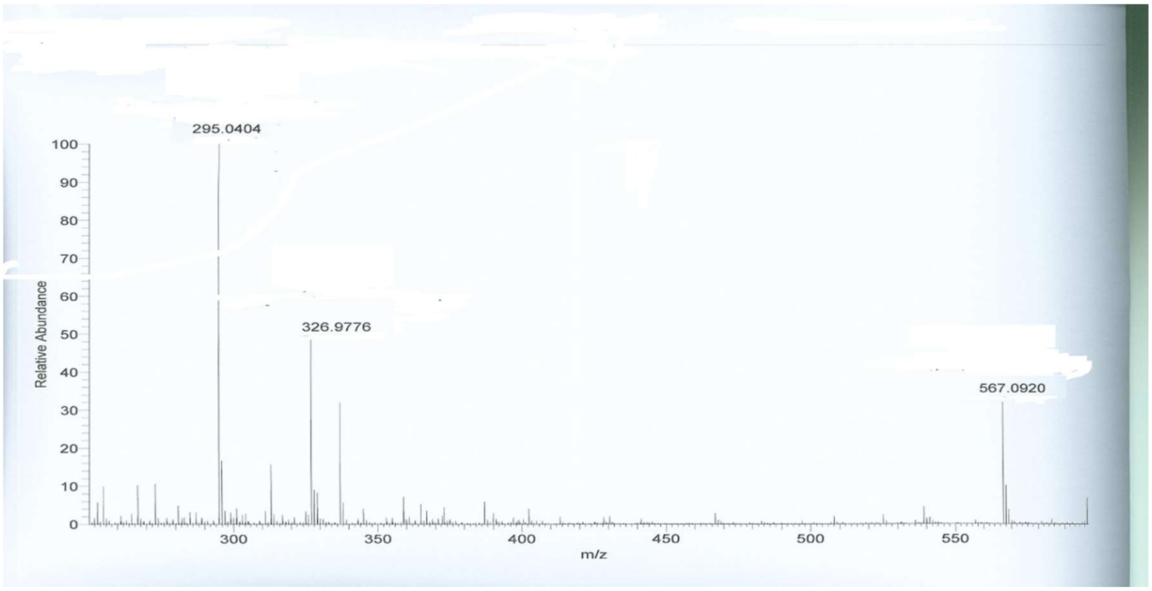

Figure N: MS spectrum of EH-3.

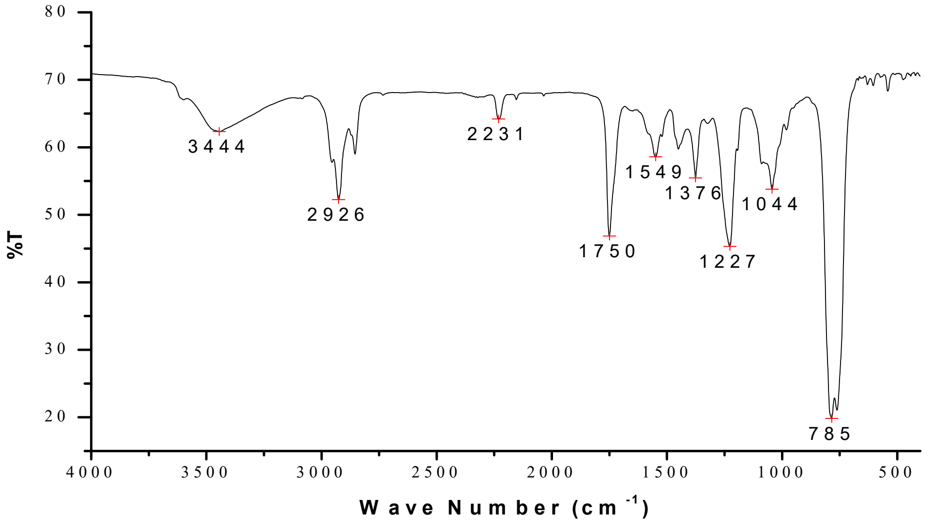

Figure O: IR spectrum of EH-3.

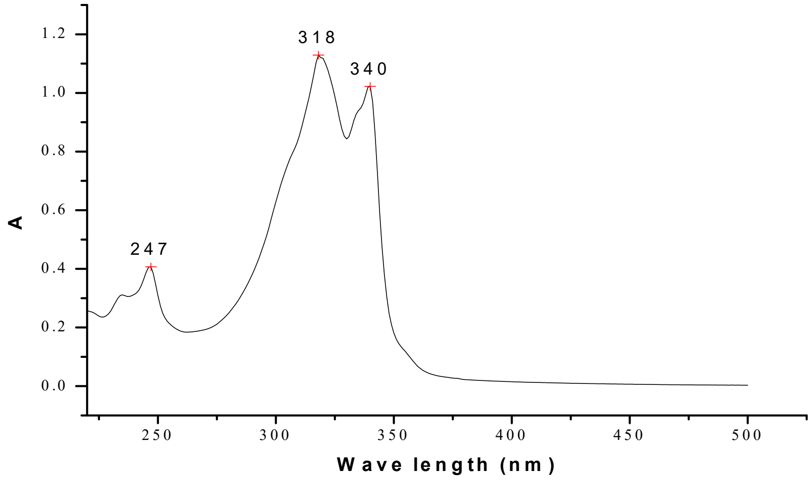

Figure P: UV spectrum of EH-3.

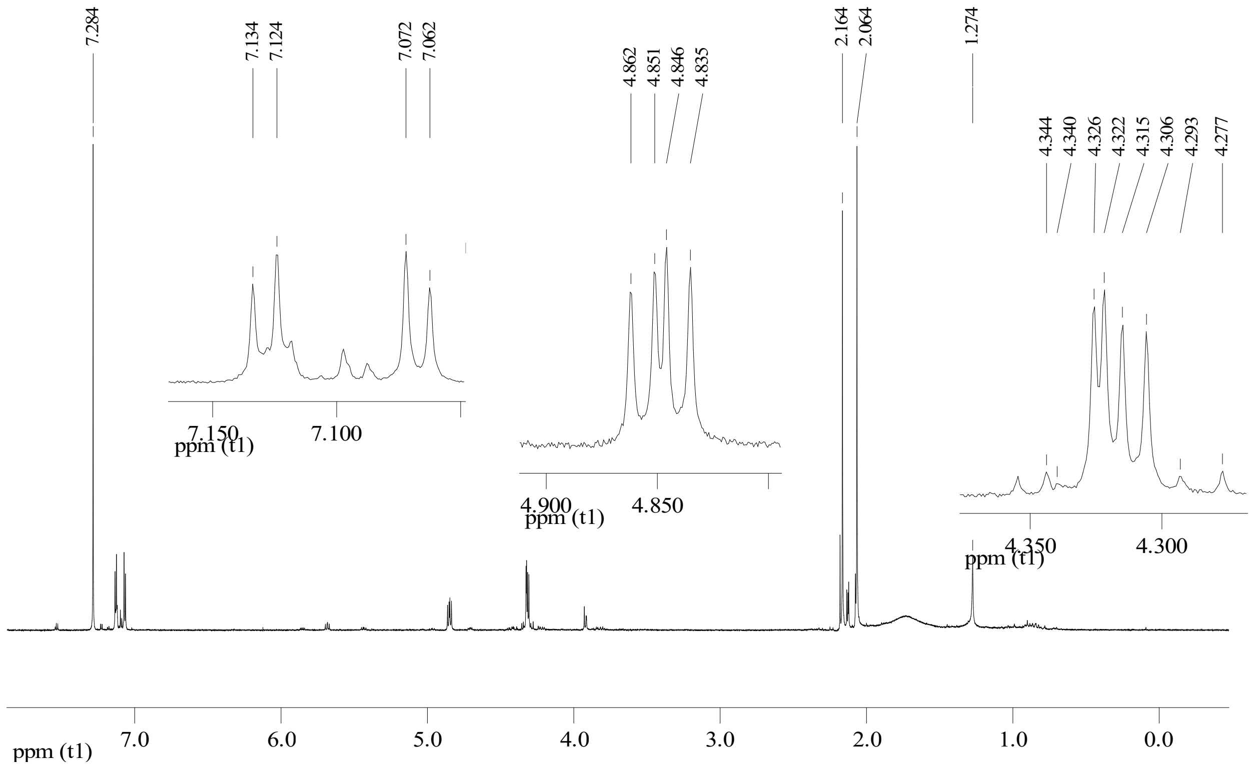

Figure Q: ^{1}H NMR spectrum of EH-3.

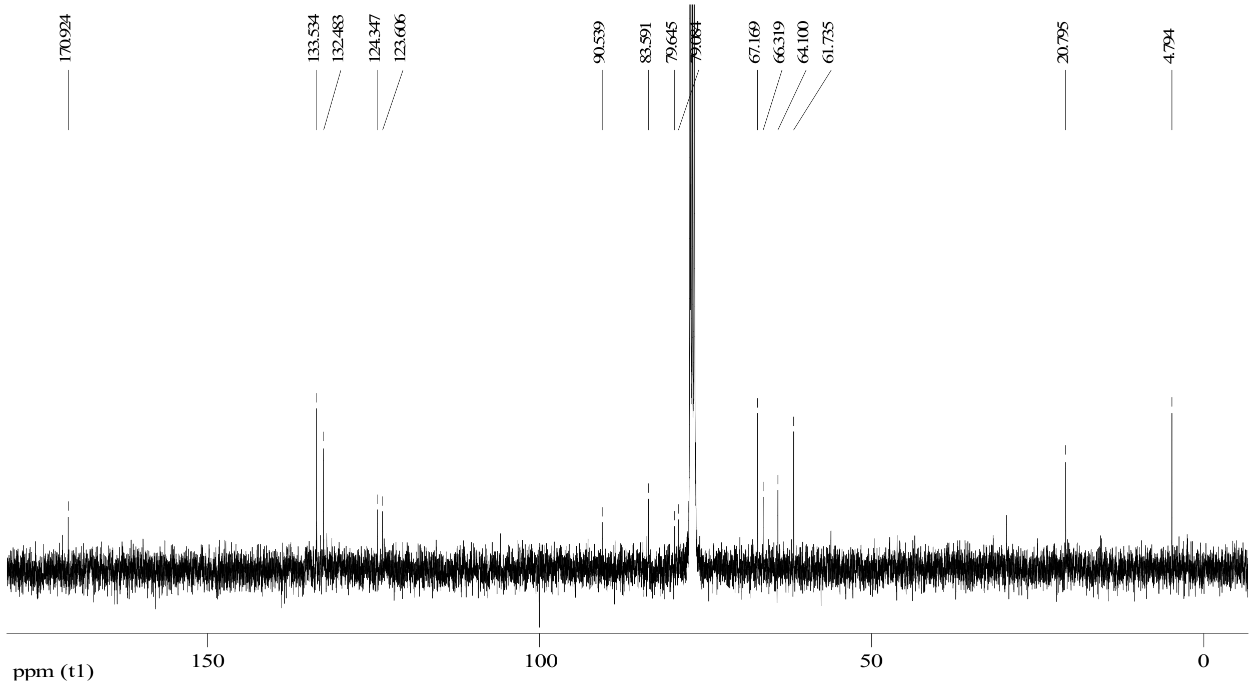

Figure R: ^{13}C NMR spectrum of EH-3.

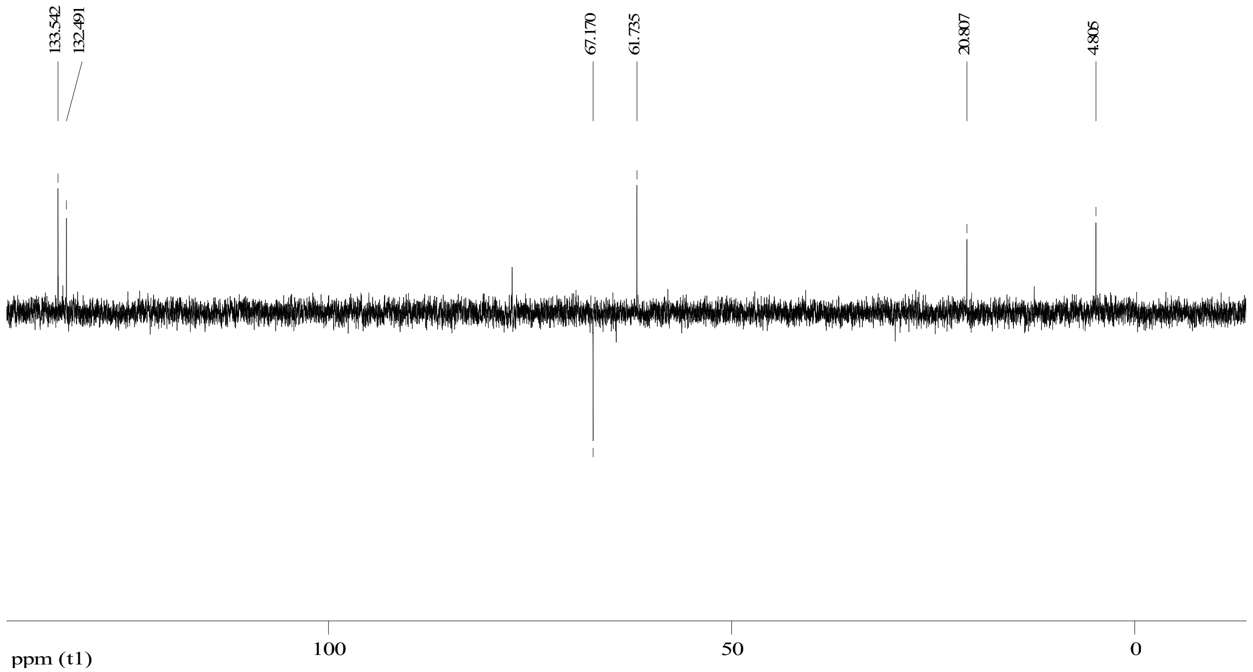

Figure S: DEPT-135 spectrum of EH-3.

Appendix V: MS, IR, UV, ^{1}H NMR, ^{13}C NMR and DEPT-135 spectra of EH-6

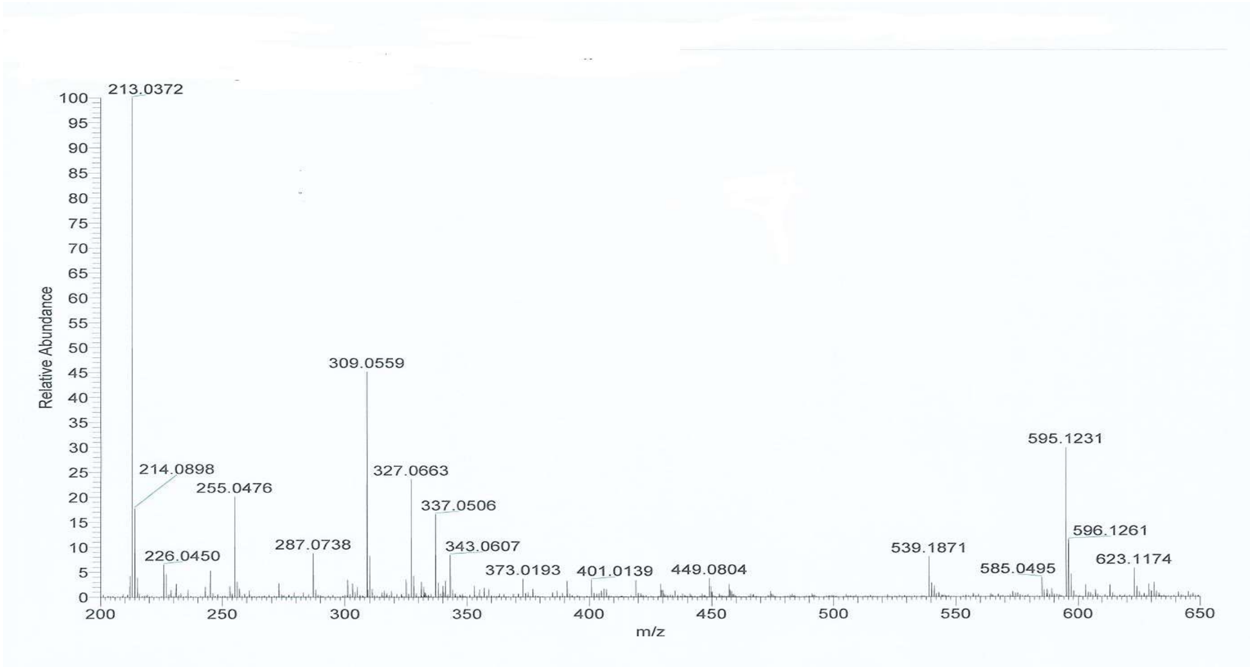

Figure T: MS spectrum of EH-6.

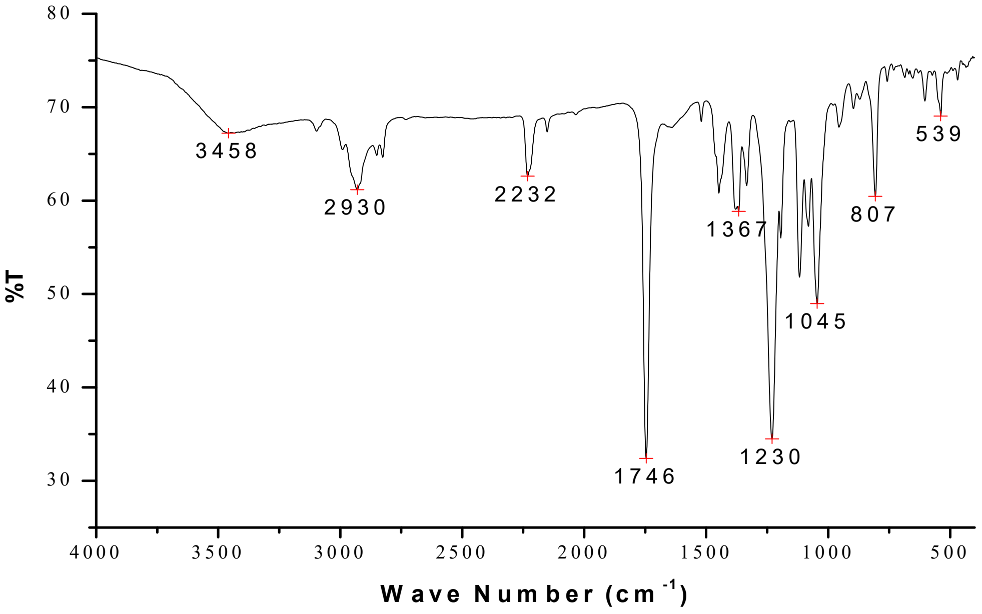

Figure U: IR spectrum of EH-6.

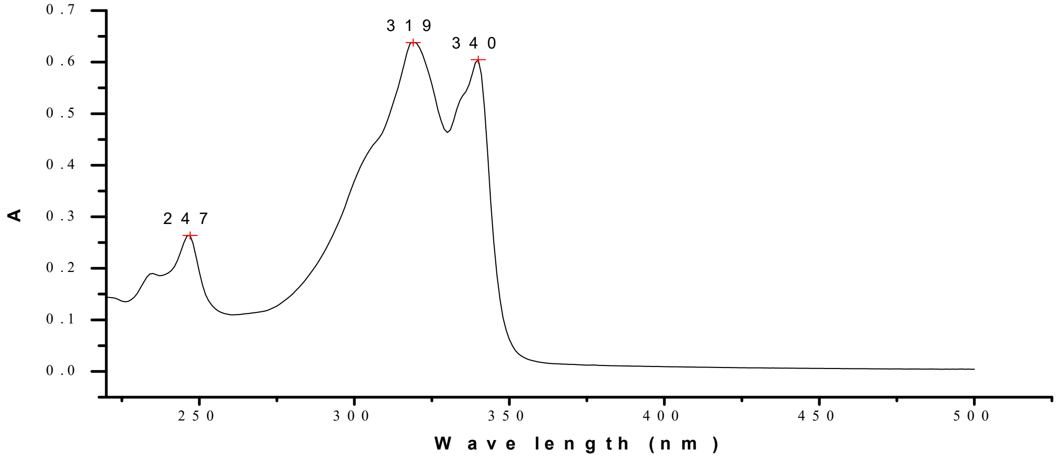

Figure V: UV spectrum of EH-6.

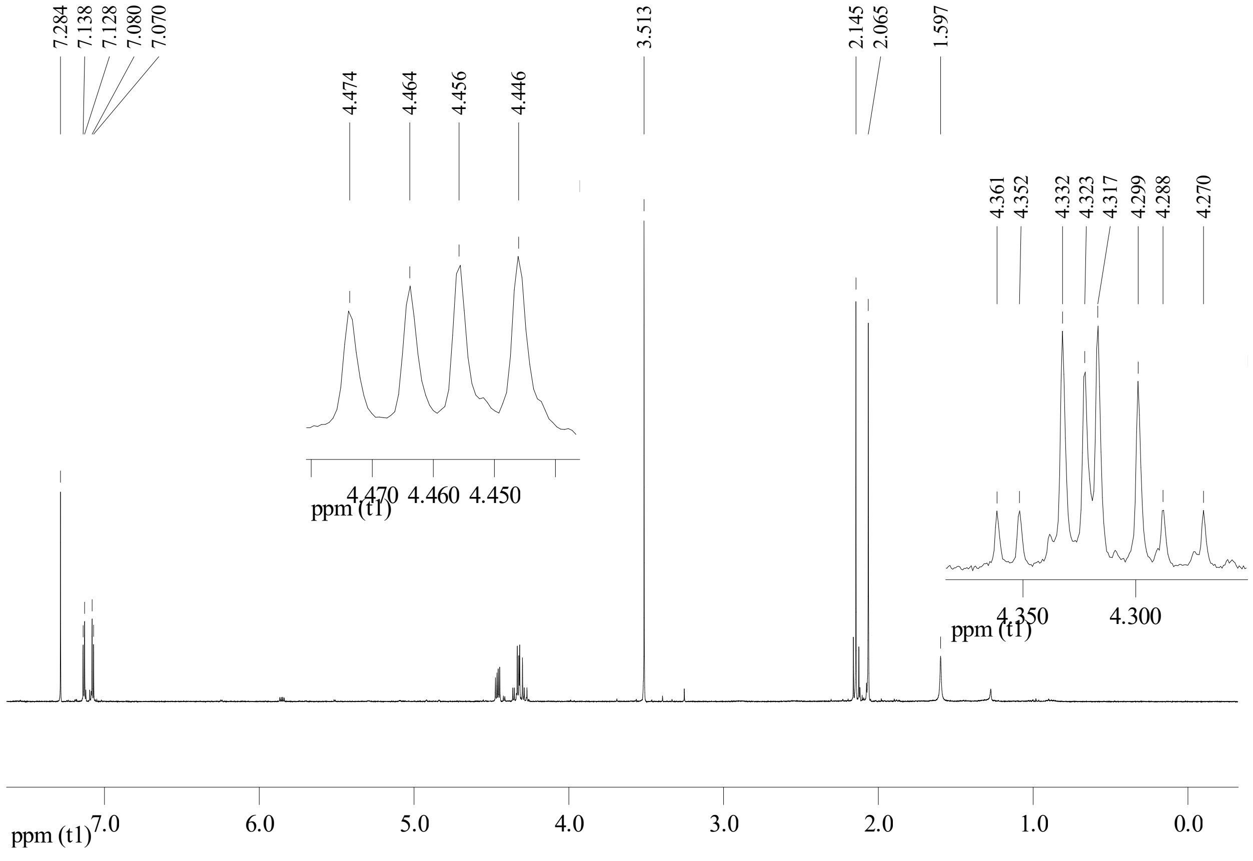

Figure W: ^{1}H NMR spectrum of EH-6.

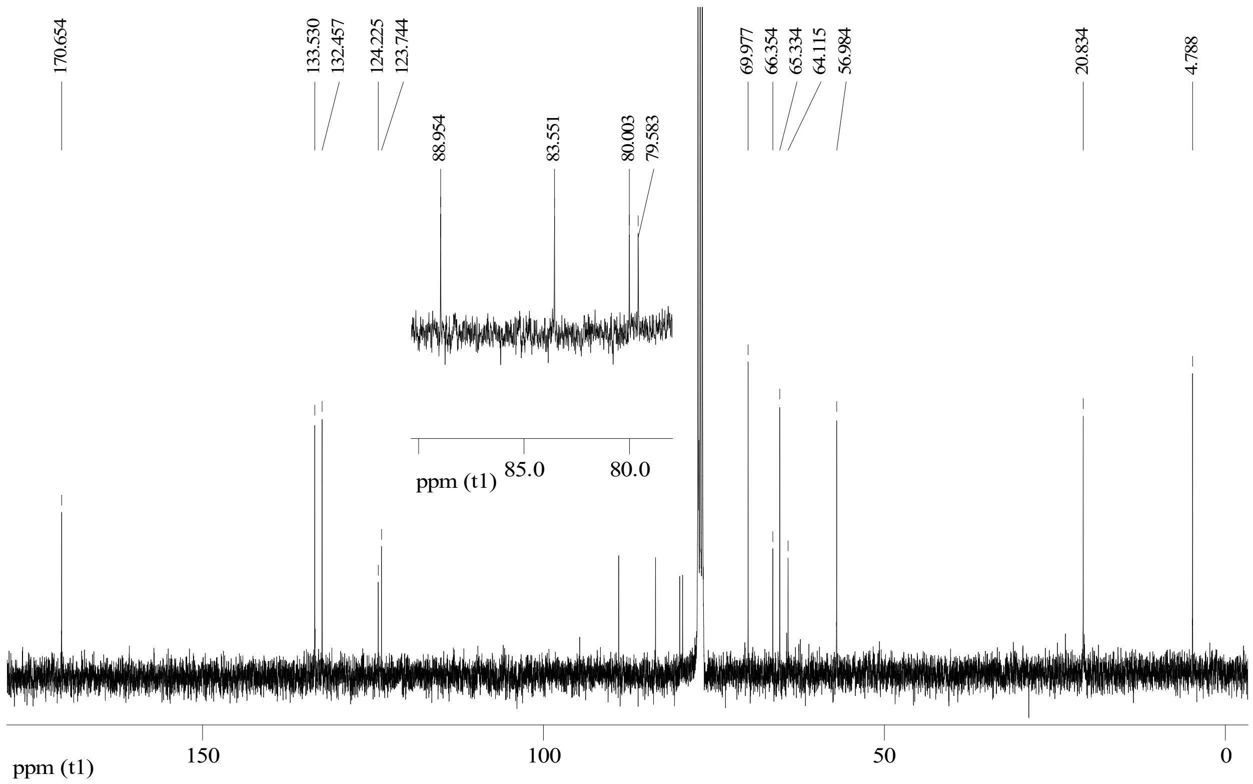

Figure X: ^{13}C NMR spectrum of EH-6.

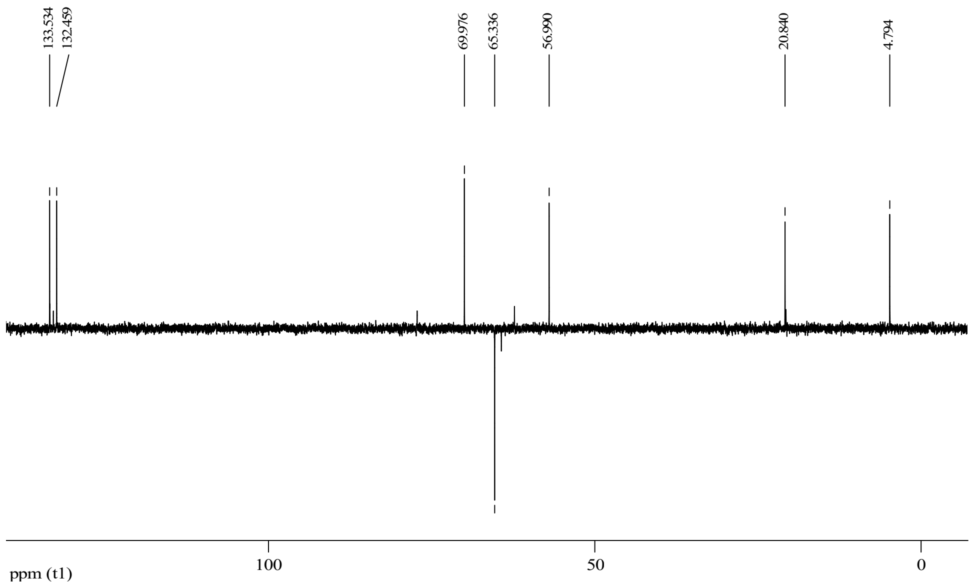

Figure Y: DEPT-135 spectrum of EH-6.

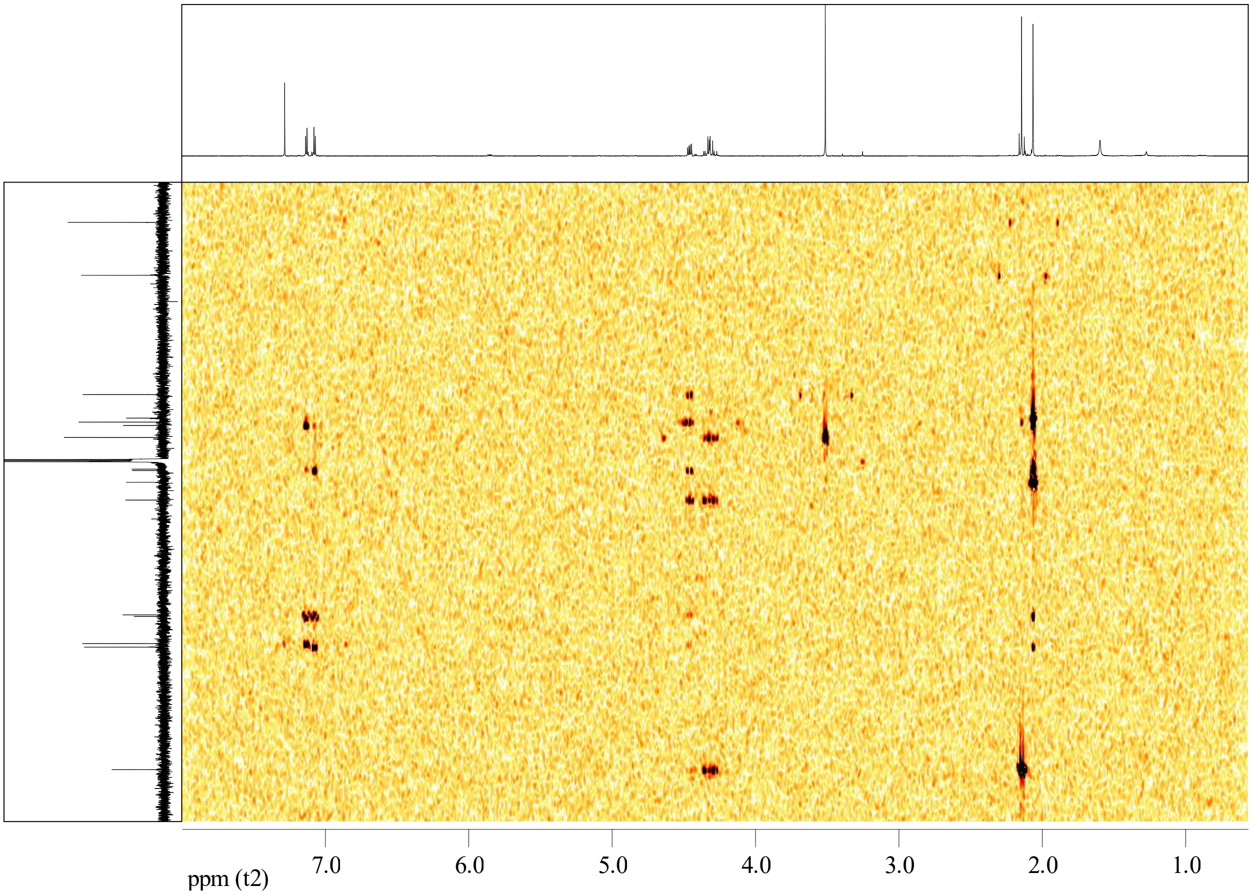

Figure Z: HMBC spectrum of EH-6.

Appendix VI: IR, UV, ^{1}H NMR, ^{13}C NMR, DEPT-135 and HMBC spectra of EH-7

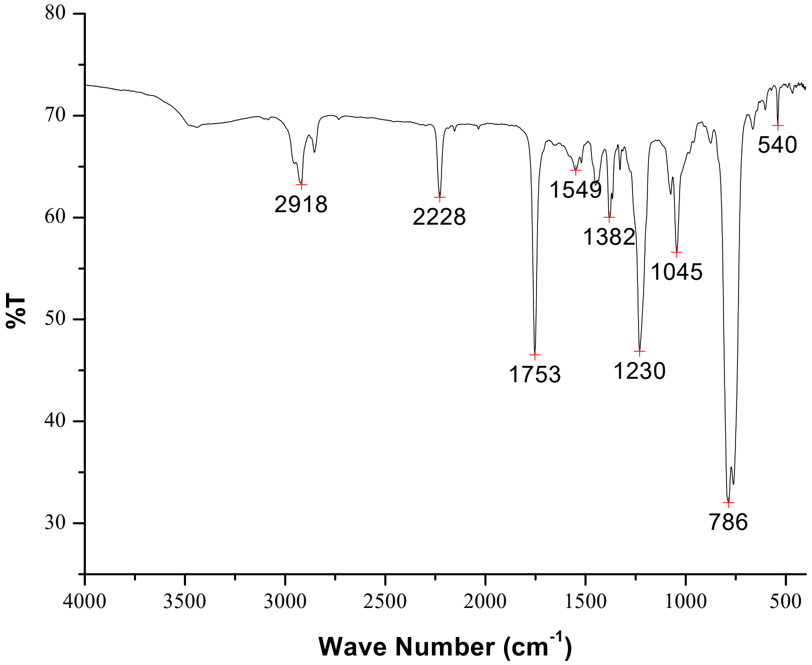

Figure A1: IR spectrum of EH-7.

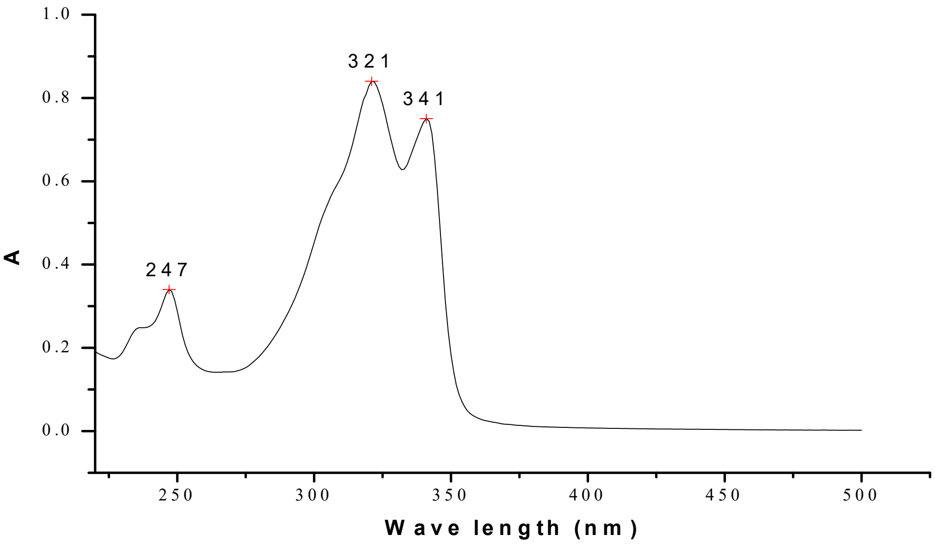

Figure B1: UV spectrum of EH-7.

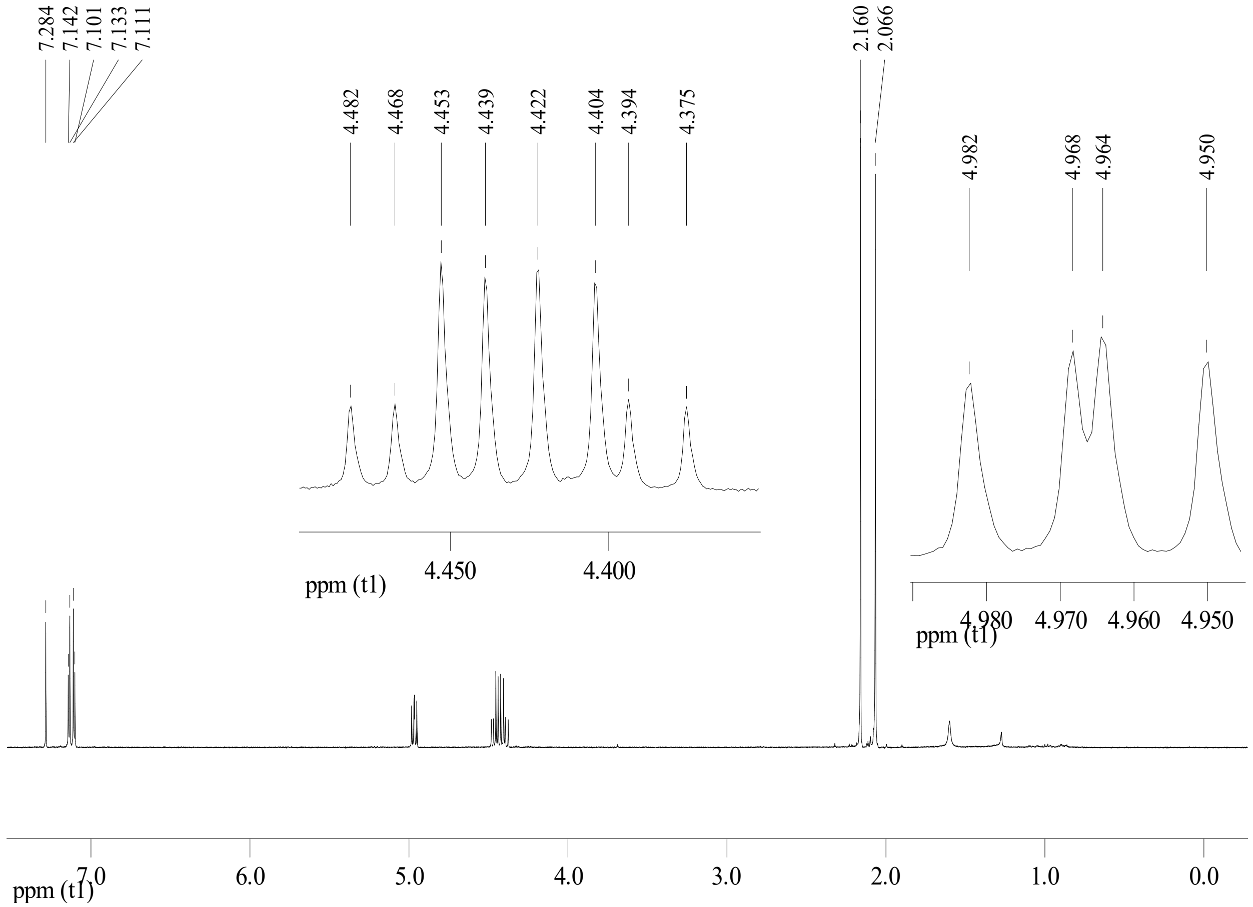

Figure C1: ^{1}H NMR spectrum of EH-7.

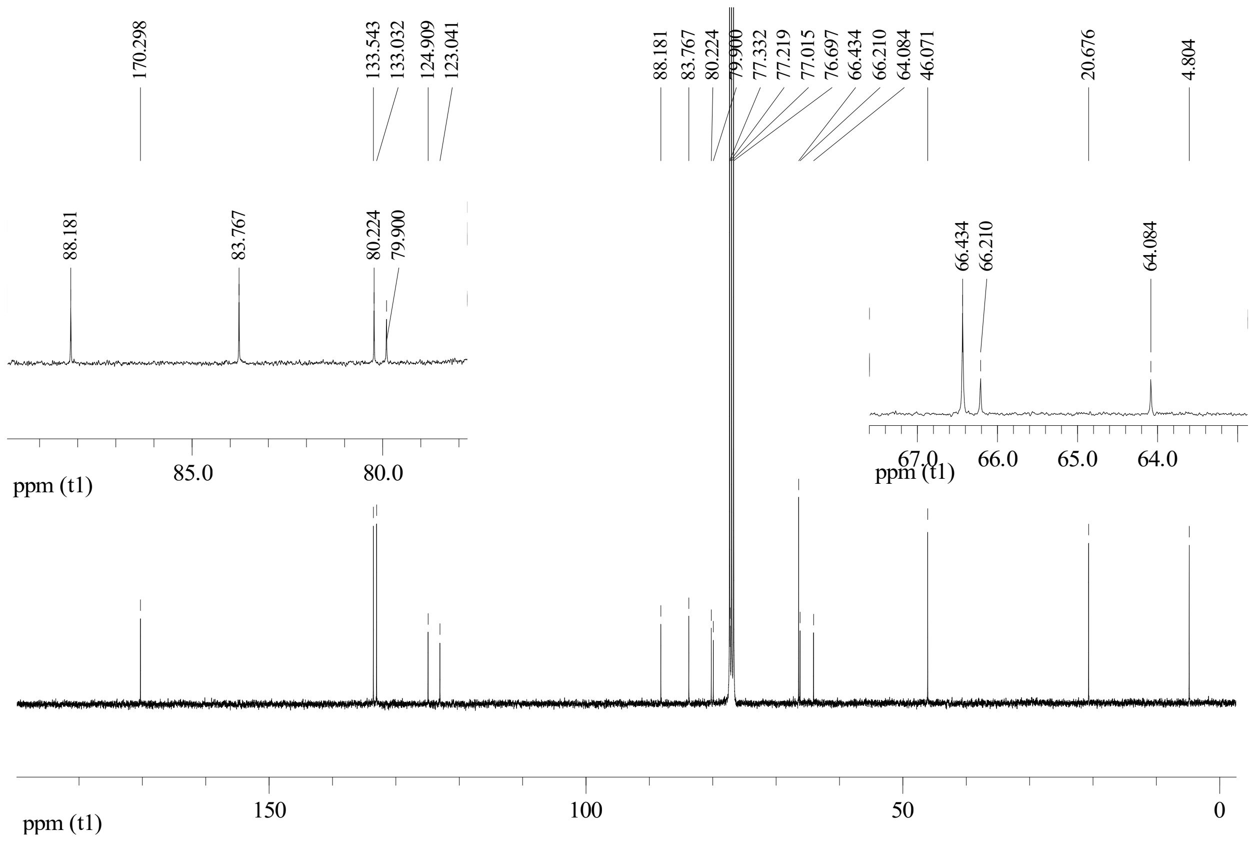

Figure D1: ^{13}C NMR spectrum of EH-7.

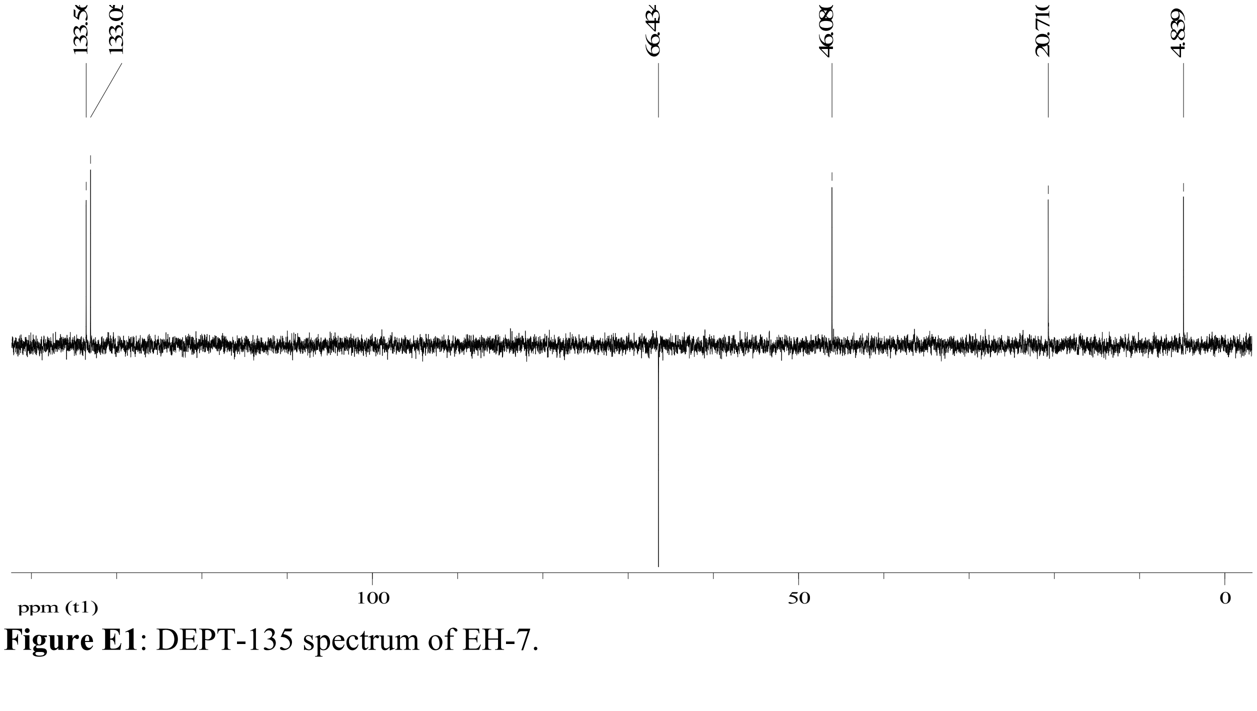

Figure E1: DEPT-135 spectrum of EH-7.

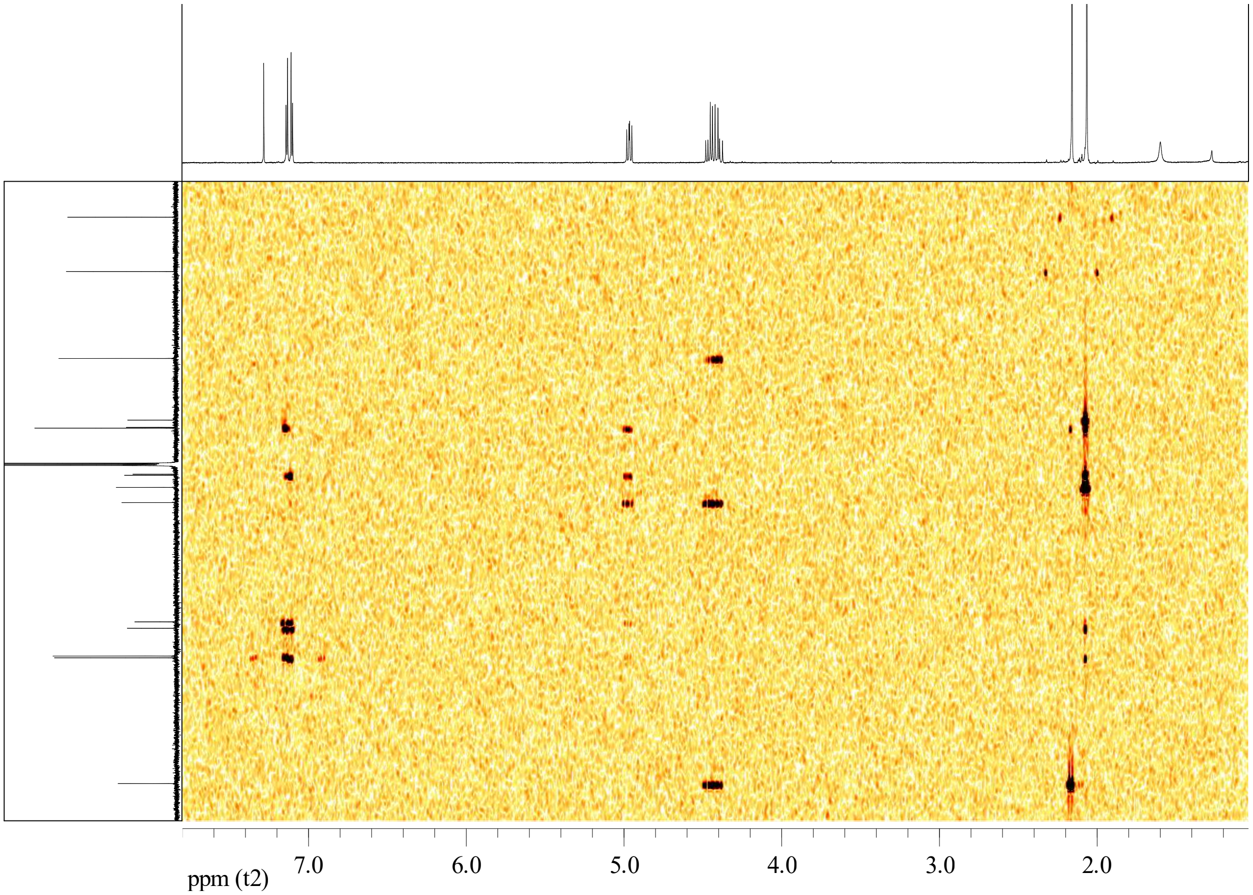

Figure F1: HMBC spectrum of EH-7.
